# Engineering Neuronal Network Connectivity Through Precise and Scalable Electrical Modulation

**DOI:** 10.1002/advs.75473

**Published:** 2026-05-07

**Authors:** Sreedhar S. Kumar, Yannaël Bossard, Rachel Sava, Tobias Gänswein, Lorenca Sadiraj, Jean‐Samuel Dupré, Manuel Schröter, J. Gray Camp, Fernando Cardes, Julian Bartram, Andreas Hierlemann

**Affiliations:** ^1^ Bio Engineering Laboratory, Department of Biosystems Science and Engineering ETH Zurich Basel Switzerland; ^2^ Ecole Normale Supérieure Paris‐Saclay Université Paris‐Saclay Gif‐sur‐Yvette France; ^3^ Institute of Human Biology (IHB) Roche Pharma Research and Early Development Roche Innovation Center Basel Basel Switzerland; ^4^ Quantitative Developmental Biology Laboratory, Department of Biosystems Science and Engineering ETH Zurich Basel Switzerland; ^5^ Biozentrum University of Basel Basel Switzerland

**Keywords:** circuit shaping, electrical stimulation, electrophysiology, hd‐mea, neuroengineering, spike timing‐dependent plasticity

## Abstract

Achieving precise and scalable control of neuronal connectivity is a key requirement for next‐generation neurotherapeutic and neuroengineering applications. Yet, implementing Hebbian‐like plasticity rules and verifying induced changes at a larger scale remain technically challenging. Here, high‐density microelectrode arrays (HD‐MEAs) are combined with programmable stimulation and analytics to induce and confirm targeted connectivity changes across multiple different in vitro and *ex vivo* preparations. Conditional Activity Metrics (CAM) are introduced to quantify stimulation‐evoked changes in relative timing and spiking density between spike train pairs. CAM changes strongly correlated with synaptic weight changes in simulations. Experimentally, we observed robust synaptic strengthening/weakening in roughly 40% of 279 tested pairs following targeted stimulation. In a subset of pairs, tracked for extended durations, the induced effects persisted for at least 90 min. Synaptic modifications were also directly validated by using simultaneous HD‐MEA and patch‐clamp recordings. This work establishes a foundation for precise, high‐throughput neuronal circuit reconfiguration, offering a versatile platform for advancing fundamental neuroscience and enabling novel neurotherapeutic and biohybrid computing strategies.

## Graphical abstract

This study presents a scalable all‐electrical method for precise neuronal‐circuit reconfiguration based on high‐density microelectrode arrays. By employing biologically inspired plasticity rules, targeted connectivity changes were successfully induced and quantified across diverse neuronal preparations. These changes persisted for extended time and were validated using simultaneous patch‐clamp recordings. The developed platform is a valuable tool for advancing neurotherapeutics and biohybrid computing.

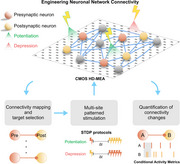

## 1 Introduction

Biological neuronal networks are inherently adaptive, continually refining their structure and function in response to experience and ongoing activity [1, 2, 3]. In the brain, this plasticity underlies learning, memory, and behavioral flexibility [4, 5, 6]. Harnessing and externally directing these adaptive mechanisms could be a powerful strategy to reconfigure neuronal circuits and, thereby, address key questions in neuroscience and neuroengineering. Such an approach would, for example, offer opportunities to probe the causal role of circuit structure in information processing [3, 7], to repair and reconfigure networks damaged by injury or disease [8, 9], and to potentially engineer efficient “wetware” computing platforms [3, 10, 11].

**FIGURE 1:**
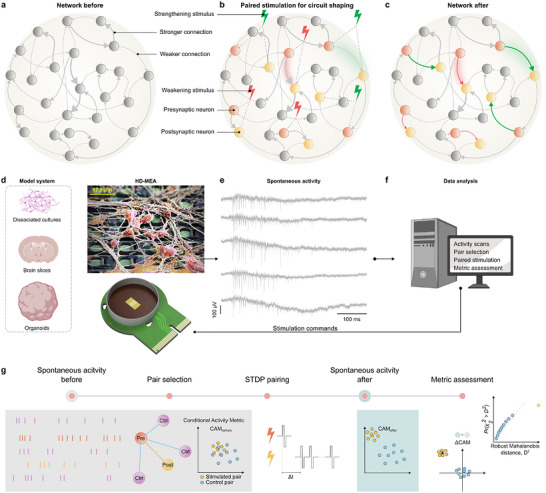
Overview of the proposed circuit shaping framework and experimental pipeline. (a–c) Conceptual schematic of a large‐scale neuronal network. Arrow thickness denotes connection strength; arrow heads indicate directionality. (b) Paired stimulation protocols can be simultaneously applied to multiple connected pairs (red bolts: weakening stimulus; green bolts: strengthening stimulus; red: presynaptic neurons; yellow: postsynaptic neurons). Pairs selected for targeted modulation are highlighted. (c) After stimulation, connections between highlighted nodes were selectively strengthened (green arrows) or weakened (red arrows) relative to baseline. (d) Schematic of the HD‐MEA platform with 26,400 electrodes at 17.5 µm pitch, interfaced with dissociated rat cortical neurons, acute mouse cortical slices, or mouse brain organoids. (top) Colorized SEM image showing rat cortical neurons cultured on an HD‐MEA (image credits: M. Oeggerli). (e) Representative raw voltage traces from five adjacent electrodes in a rat cortical culture, showing spontaneous activity. (f) The hardware platform is complemented by a customized data analysis pipeline that provides user‐defined control over electrode selection, stimulation timing, and other protocol parameters. (g) Experimental and analysis workflow. Candidate pre–post electrode pairs are identified based on spontaneous activity. Each trial consists of a single biphasic pulse at the presynaptic electrode, followed by a variable delay (Δt, –10 to +10 ms) and a burst of stimulations at the postsynaptic electrode. Protocols are delivered in single‐ or multi‐pair mode. Post‐stimulation changes in Conditional Activity Metrics (CAM) are quantified as ΔCAM (see Section 2.3). Stimulated pairs (yellow) are then compared to a null distribution obtained from control electrodes (blue) in the same network. Functional changes are quantified using the robust Mahalanobis distance between the respective distributions.

Hebbian‐like learning rules provide a natural framework for inducing and steering adaptive changes [12]. In particular, the concept of spike timing‐dependent plasticity (STDP) – where precise temporal correlations between pre‐ and postsynaptic spikes drive long‐lasting changes in synaptic efficacy – offers a mechanistic basis for reconfiguring existing neuronal connections ([13, 14, 15, 16]; Figure 1a–c). However, experimentally inducing selective and directed connectivity changes at scale is still constrained by methodological limitations.

Existing experimental approaches provide valuable insights into plasticity mechanisms but lack the combination of precision, selectivity, and scalability required for connectivity manipulations at the network level. The patch‐clamp technique – often involving multiple electrodes [13, 14] or combined with extracellular stimulation [17, 18] – has typically been used to study STDP in biological neuronal networks. It permits the unambiguous assessment of preexisting connections, precise stimulation control, and verification of induced synaptic changes. However, the technique is invasive, which compromises cell viability, and is also technically demanding. Moreover, it is inherently low throughput, thus limiting studies to single individual connections over relatively short periods. All‐optical methods offer precise and flexible access to multiple loci [19, 20]. However, the samples must be genetically modified, and the method lacks scalable readouts for identifying existing connections and assessing intervention outcomes [20]. While studies on low‐density microelectrode arrays have used paired electrical stimulation to investigate network plasticity [21, 22], the limited spatial resolution of these systems precludes precise interventions and detection of induced effects and has hindered a direct demonstration of controlled large‐scale plasticity. Achieving scalable circuit shaping requires non‐invasive, connection‐specific control of spike timing and robust verification of the induced changes.

To address the issues outlined above, we introduce a hardware‐enabled experimental and analytical pipeline that allows for precise and scalable circuit shaping in biological neuronal networks. Our core innovation lies in synergistically combining complementary metal‐oxide‐semiconductor (CMOS) high‐density microelectrode array (HD‐MEA) technology [23, 24, 25, 26] with scalable analytical routines in an iterative workflow (Figure 1d–g). HD‐MEAs provide stable, noninvasive, and bi‐directional access to thousands of neurons over extended durations. This capability makes precise and parallel network manipulation possible while simultaneously allowing for the observation of large‐scale network activity. HD‐MEA readouts enable the functional inference of preexisting connections and guide the selection of candidate target sites for intervention. Temporally precise, patterned, and spatially confined stimuli are then delivered to the target sites, and, in selected cases, their efficacy is verified using directly evoked responses obtained through a custom artifact‐suppression routine.

To enable rigorous and scalable verification of induced connectivity changes, we developed a data‐driven analytical approach and a quantitative measure – Conditional Activity Metrics (CAM). This model‐free measure quantifies changes in spike‐train relationships following patterned stimulation, in particular, shifts in the conditional latency of spike occurrence and the spiking frequency. By enabling a rapid, spike train‐based assessment of induced plasticity, CAM provides a reliable, high‐throughput alternative to invasive verification techniques.

We demonstrate the effectiveness of our platform across diverse in vitro (rat primary dissociated cortical cultures and stem‐cell‐derived brain organoids) and *ex vivo* (acute mouse cortical slices) neuronal preparations, establishing a general framework for externally guiding plasticity in neuronal circuits. Our platform provides a validated all‐electrical approach to network‐scale engineering, enabling parallel and selective sculpting of network architectures. We anticipate that this approach will open new avenues for understanding and manipulating the functional repertoire of adaptive neuronal systems.

## 2 Results

### 2.1 HD‐MEA‐Based Platform for Targeted Circuit Shaping

We developed a CMOS HD‐MEA‐based platform that enables the measurement and modulation of neuronal network activity at subcellular spatial resolution and high temporal resolution. The core of the platform is an HD‐MEA, previously described by Ballini et al. [23], which we integrated into a workflow for targeted stimulation and network‐level circuit shaping (Figure 1d). The array featured 26,400 electrodes covering a sensing area of approximately $3.85\times 2.10{\mathrm{mm}}^{2}$ at an electrode pitch of 17.5 µm. Up to 1024 readout channels could be flexibly configured for simultaneous voltage recordings at a sampling rate of 20 kHz. Integrated on‐chip stimulation units delivered programmable pulses to user‐defined sites, supporting diverse paired‐stimulation protocols for activity‐dependent plasticity induction. A reconfigurable switch matrix enabled dynamic routing of recording and stimulation units, allowing virtually unrestricted targeting of neuron pairs and adaptation to the morphology of diverse biological preparations (Figure 1d,e). Together with customized, data‐driven routines that interface directly with the chip, the hardware and software components formed an integrated pipeline offering user‐defined control over electrode selection, stimulation timing, and protocol parameters (Figure 1f).

The workflow began with a spatial scan of spontaneous activity across the array electrodes to localize active regions, followed by the selection of informative recording sites. Spontaneous spiking was monitored at these sites for 15 to 45 min (Figure 1e) to establish baseline connectivity profiles (Figure  S6).

Targeted circuit modulation builds on STDP, an asymmetric synaptic learning rule in which the relative timing of pre‐ and postsynaptic spikes determines the polarity and magnitude of changes in synaptic efficacy [13, 15]. Because effective plasticity induction requires preexisting connections, a preselection pipeline identified candidate electrode pairs with access to putatively connected neurons (see Section 4.9; Figure S6). Here, “pre‐”and “postsynaptic sites” refer to electrodes that delivered the respective components of the paired stimulation protocol. The pairing paradigm typically consisted of a biphasic presynaptic pulse followed by a burst of 510to pulses at the postsynaptic site (see Section 4.6; Figure 1g). This pre–post burst protocol, previously shown to be effective in eliciting long‐term potentiation and long‐term depression [27], can be applied repetitively to single pairs or simultaneously to multiple selected pairs. Programmable delays between pre‐ and post‐stimulation components, typically ranging from ‐10 to +10ms‐, bias the outcome toward potentiation or depression (Figure 1g).

Following stimulation, the platform monitored ongoing spontaneous activity for an additional 15 to 30 min. Stimulation‐induced changes were quantified based on Conditional Activity Metrics (CAM; Figure 1g; see also Section 2.3 and Section S1.1). Statistical comparisons between stimulated pairs and non‐stimulated controls in the same networks provided a robust assessment of the efficacy and specificity of targeted circuit shaping.

### 2.2 Stimulation Precision, Efficacy, and Targeted Plasticity Induction

Spatially resolved plasticity induction is contingent upon the precise and reliable delivery of spatiotemporally patterned stimulation protocols. As little data exist on whether the stimulation capabilities of modern HD‐MEAs can meet the corresponding requirements, we systematically validated that our HD‐MEA stimulation protocols (1) reliably evoked biologically relevant responses, (2) remained spatially localized, (3) operated with low‐millisecond temporal precision, and (4) effectively induced synaptic plasticity.

**FIGURE 2:**
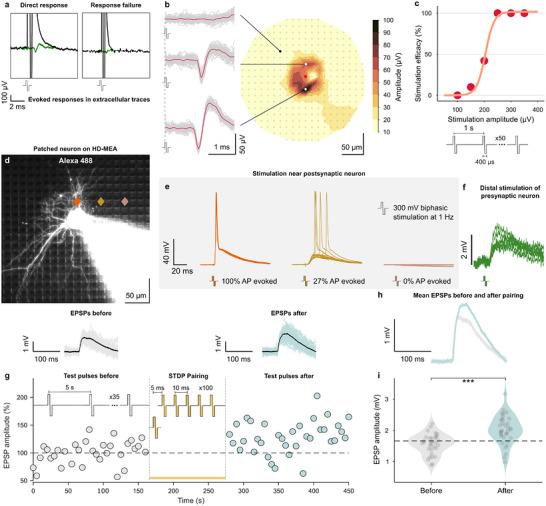
Validation of stimulation precision, efficacy, and targeted plasticity induction. (a) Raw extracellular traces (black) recorded from an electrode adjacent to the stimulation site during single‐pulse stimulation (350 mV/phase, 200 μs/phase) in an acute mouse cortical slice. Stimulation artifacts obscure direct neuronal responses. Artifact‐suppressed traces (green), revealed successfully evoked responses (left) or response failures (right). (b) Spatial map of direct response amplitudes (color scale in μV) across the HD‐MEA, demonstrating direct response localization within a 35 μm radius of the stimulation site (red dot; 350 mV/phase, 1 Hz). Left: Overlaid responses from 50 trials at the marked electrodes illustrating response reliability; red traces indicate the mean. (c) Stimulation efficacy as a function of amplitude (100 to 350 mV/phase), showing the threshold for reliable responses (>300 mV/phase; n=50 trials/amplitude). (d) Representative postsynaptic neuron in a rat primary dissociated cortical culture filled with Alexa Fluor 488 via the patch pipette to visualize its morphology against the HD‐MEA chip background. Stimulating electrodes proximal to the soma are indicated by diamonds. (e) Only peri‐somatic stimulation reliably evoked postsynaptic action potentials, demonstrating spatial precision of the selected stimulation parameters (15 trials per site). (f) Stimulation of more distal surrounding neurons, identified through spontaneous activity, revealed a putative monosynaptic connection to the patched cell (green traces; 8 trials). (g) Excitatory postsynaptic potential (EPSP) amplitudes before (gray; ∼3 min) and after (teal; ∼3 min) application of a spike timing–dependent plasticity (STDP) pairing protocol (100 repetitions). Each dot represents a single AP‐evoked EPSP, normalized to baseline. Overlaid traces (above) show representative EPSPs before (gray) and after (teal) pairing; a black trace indicates the mean. (h) Average unitary EPSPs before and after pairing, illustrating a 20% increase in amplitude. (i) Distribution of individual EPSP amplitudes (mV) before and after pairing demonstrates a significant potentiation (t(65)=−4.66, P<0.001; left‐tailed two‐sample t‐test; $n_{1}=32$, $n_{2}=35$). All stimuli were biphasic voltage pulses of 300 mV/phase. Responses overlapping with spontaneous network activity were excluded. Similar results were reproduced in three additional patched cells.

To assess whether direct responses were reliably evoked, we delivered single biphasic pulses (amplitudes between 100 to 350 mV/phase, 200 μs/phase) at 1 Hz to individual electrodes on the HD‐MEA in acute mouse cortical slices. Raw traces from neighboring channels exhibited prominent stimulation artifacts that obscured direct neuronal responses (Figure 2a, black trace), a commonly known limitation of electrical stimulation on HD‐MEAs [28]. To address this issue, we developed a dedicated artifact suppression pipeline based on hybrid local regression (see Section 4.14), which allowed us to unambiguously identify direct responses (Figure 2a; green trace, left) and response failures (Figure 2a; green trace, right). Direct responses overlaid from 50 stimulus trials (Figure 2b, left) confirmed that evoked responses were highly reliable for stimulation signal amplitudes exceeding 300 mV/phase, which were used for most experimental pairs (Figure 2c). To assess the spatial confinement of the stimulation response, we computed a spatial map of direct responses and found that it was confined to electrodes within a 35 μm radius of the stimulation site (Figure 2b; stimulation amplitude: 350 mV/phase). This spatial confinement suggested that only a limited subset of neurons near the stimulation site were directly activated. However, consistent with the known properties of extracellular stimulation, the potential occurrence of antidromic co‐activation of distal neurons via axons of passage cannot be entirely excluded.

To verify the temporal precision of our dual‐site, paired stimulation protocol for STDP induction, we delivered a single pulse to a selected presynaptic site, followed 5 ms later by a 5‐pulse 100 Hz train at the postsynaptic site [27](see Section 4.9 for the selection procedure of electrode pairs). Analysis of artifact timing confirmed the temporal precision: the observed pre‐post delay was 5.4 ms (accounting for the 0.4 ms pulse duration), and the inter‐pulse interval was ≈10.02 ms, both closely matching the intended values. Unfiltered traces from electrodes adjacent to the stimulation sites, after applying our dedicated artifact suppression pipeline, revealed time‐locked direct responses confined to the respective sites, again confirming the spatial specificity of the stimulation protocol (Figure S1).

While the temporal precision and spatial localization of extracellular stimulation could be considered similar across preparations – since the dominant sources of delay stem from the electronics – biological and experimental variability introduced heterogeneity in response probability. Thus, the reported efficacy values should be interpreted as representative but not exhaustive across all stimulated units.

To validate the induction of synaptic plasticity following the HD‐MEA‐based stimulation protocol, we performed simultaneous whole‐cell patch‐clamp recordings on selected postsynaptic neurons in rat primary dissociated cortical cultures. A representative example of a patched postsynaptic neuron at 20 days in vitro, filled with Alexa Fluor 488 via the patch pipette, is shown in Figure 2d.

Using the whole‐cell current‐clamp mode, the neuron was first localized on the HD‐MEA (see Section 4.7). To assess the reliability of extracellular action potential induction in the patched cell, we sequentially applied biphasic voltage stimuli to different HD‐MEA electrodes near the cell soma at 1 Hz. Only perisomatic stimulation reliably evoked postsynaptic action potentials (Figure 2e; 15 trials per site), in line with previous studies involving electrical stimulation on HD‐MEAs [28, 29].

To probe for connected presynaptic neurons, we first performed a brief assessment of spontaneous network spiking activity and then stimulated putative presynaptic neurons, choosing the electrode that exhibited the largest signal amplitude in the extracellular neuronal footprint as the stimulation site [30]. A putative excitatory monosynaptic connection to the patched neuron was identified when a probing stimulus (at 1 Hz) at the presynaptic site exhibited a correlated excitatory postsynaptic potential (EPSP) in the patched cell (Figure 2f; 8 trials). The identified pre‐ and postsynaptic stimulation sites were then used for the subsequent STDP experiment.

The application of an STDP pairing protocol (100 repetitions) induced robust potentiation of unitary EPSPs, as shown by the 20% increase in mean EPSP amplitudes post‐pairing (Figure 2g,h). The distribution of EPSP amplitudes before and after pairing demonstrated significant potentiation (t(65)=−4.66, P<0.001; left‐tailed two‐sample t‐test; $n_{1}=32$, $n_{2}=35$; Figure 2i). These results were reproduced in three additional patched cells, confirming the biological efficacy of our stimulation protocol.

Together, these results demonstrate that our HD‐MEA stimulation system reliably and precisely evoked localized neuronal activity and induced biologically relevant synaptic plasticity. The combination of artifact suppression, spatial mapping of direct responses, and patch‐clamp validation ensured the methodological robustness of our workflow and provided a solid foundation for targeted circuit shaping.

### 2.3 Conditional Activity Metrics (CAM)

A robust platform for manipulating neuronal connectivity requires methods that directly verify sustained changes in coupling strength. Patch–clamp assays provide verification; however, they are invasive and have low throughput. Spike train–based functional connectivity metrics offer a scalable alternative, but only capture static relationships and lack statistically rigorous formulations for detecting connectivity changes between pre‐ and post‐intervention epochs [31, 32, 33, 34] (see also Figure S8).

We, therefore, introduce CAM, a spike train‐based measure that quantifies systematic changes in synaptic strength through the statistical comparison of pre‐ and post‐intervention spike train pairs. We hypothesize that selective synaptic enhancement causes postsynaptic spikes (train j) to occur (a) with shorter latencies and (b) with higher conditional probabilities following presynaptic spikes (train i), with the opposite pattern expected for synaptic depression [35, 36, 37]. We capture these attributes in a pair‐wise, two‐dimensional, probabilistic measure:

(1) $${\mathrm{CAM}}^{i,j}=\left(\begin{array}{c} t_{1}^{i,j}\\ \rho^{i,j} \end{array}\right).$$

**FIGURE 3:**
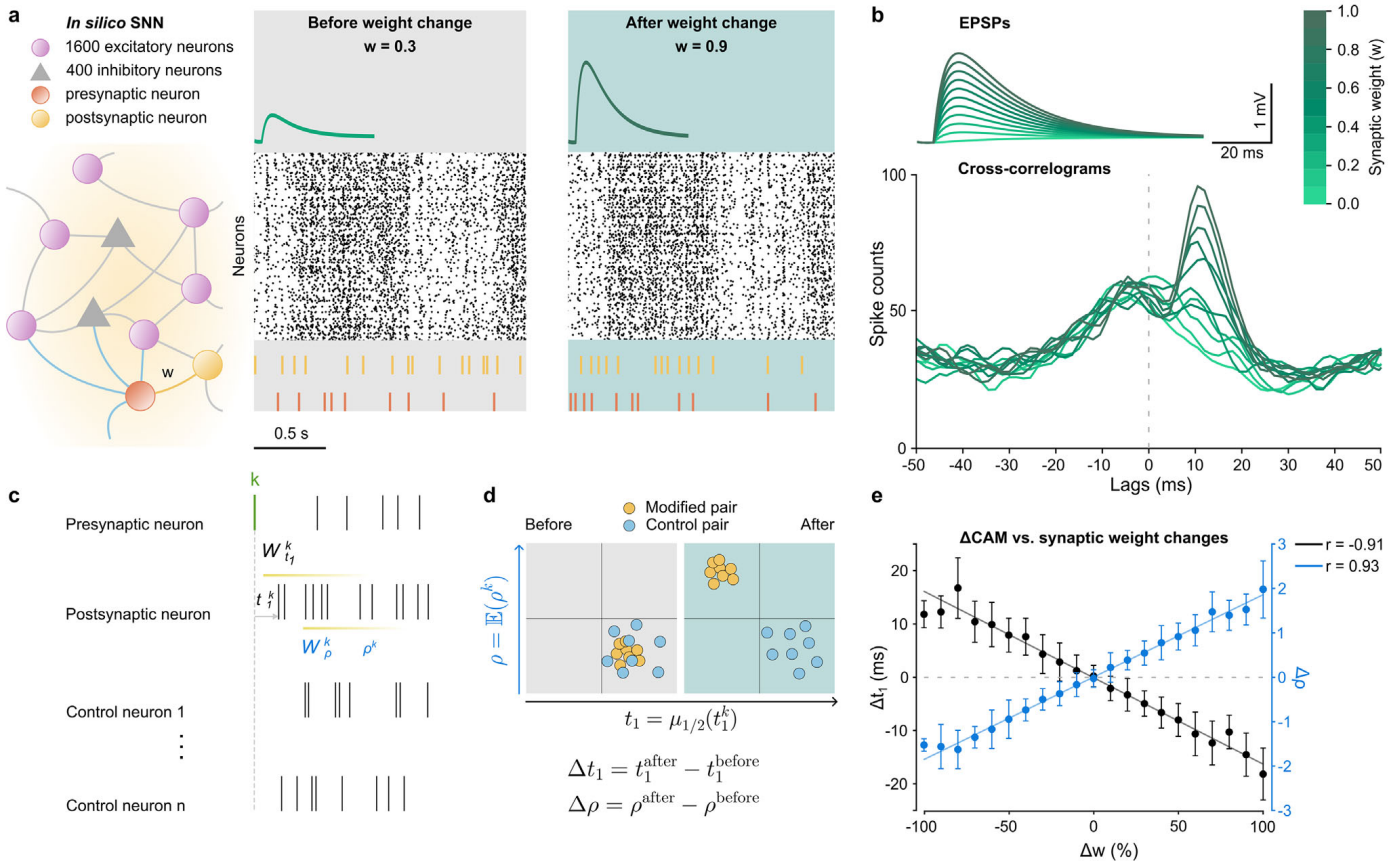
Evaluation of Conditional Activity Metrics (CAM) in in silico spiking neural networks. (a) CAM was evaluated by selectively modifying synaptic weight (w) and delay (Δt) between selected pre‐ and post‐synaptic excitatory neuron pairs colored in red and yellow in simulations of 2000‐neuron networks. The raster plot shows a few seconds of activity of the 1600 excitatory neurons in the network. (b) Increased w induced stronger EPSPs (top) and distinct peaks in the cross‐correlograms (bottom). Binned cross‐correlograms were computed for multiple synaptic weight changes for the same selected pair of neurons. (c) CAM quantifies latency to the first spike ($t_{1}$) and spike density (ρ) conditional on presynaptic spikes, computed for defined post‐spike windows. (d) CAM values shifted after synaptic weight changes (gray: baseline; teal: after change); yellow and blue markers denote CAM for modified and control neuron pairs, respectively. Variability of CAM for the selected pair was assessed via spike‐time resampling (yellow cluster). (e) Across simulations, Δρ and $\Delta t_{1}$ were strongly correlated with weight changes ($r_{\Delta \rho }\left(997\right)=0.93$; $r_{\Delta t_{1}}\left(997\right)=-0.91$; P<0.001 for both), confirming sensitivity of the metric to synaptic weight changes.

Here $t_{1}^{i,j}$ is the first‐spike latency in j within a prescribed post‐spike search window $W_{t_{1}}$ (reported as the conditional median across spikes in the reference train i), and $\rho^{i,j}$ is the conditional spike density of j within a (possibly distinct) post‐spike window $W_{\rho }$ (reported as the conditional expectation; Figure 3c). Thus, CAM describes the distribution of postsynaptic timing and firing rate dynamics for given presynaptic events (see Section S1.1 for a formal definition).

The change in CAM of a given pair of spike trains, ΔCAM 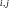, before and after the intervention, captures alterations in both the temporal structure ($\Delta t_{1}$: change in first spike latency) and firing rate dynamics (Δρ: change in spike density) of postsynaptic responses (Figure 3d). Together, it provides an indirect measure of synaptic plasticity  (Equation S5). In general, synaptic potentiation is reflected in shorter latencies ($\Delta t_{1}<0$) and higher spike densities (Δρ>0), whereas depression manifests as the converse.

To characterize the sensitivity of CAM features to changes in synaptic strengths, we used in silico spiking neural network simulations with explicitly defined synaptic weights and delays (Figure 3a). Manipulating individual synapses produced predictable changes in excitatory postsynaptic potentials and corresponding binned spike cross‐correlograms (Figure 3b). CAM captured these changes with high fidelity: changes in synaptic weight were tightly correlated to latencies and densities (Figure 3d–e; Δρ:r(997)=0.93, $\Delta t_{1}:\;r\left(997\right)=-0.91,\;P<0.001$ for both). Moreover, CAM changes exhibited the strongest correlation to synaptic weight changes, outperforming existing spike train–based functional connectivity metrics under identical conditions (see Section S1.4; Figure S8).

Importantly, CAM changes may not always follow the simple patterns observed in our controlled simulations. Shifts in CAM do not necessarily occur along both axes simultaneously (Figure S5). A potentiated connection that contributes early during cooperative postsynaptic firing can strongly and reciprocally influence both first‐spike latency and spike density. If it contributes later, it may primarily elevate spike density without appreciably altering latency. Conversely, depression may manifest as prolonged latency or reduced density, depending on the relative timing and contribution. In cases where recurrent drive is weak, an exclusive change in latency may arise without a corresponding density effect. Given this complexity, rather than interpreting individual CAM components in isolation, we quantified whether stimulated pairs exhibited a statistically significant net displacement in the CAM plane relative to control pairs to infer a stimulus‐induced effect.

Because apparent CAM shifts may also arise spontaneously from changes in ongoing network activity, it is essential to identify stimulus‐induced effects not only across temporal windows but also in the context of concurrently evolving network activity. To this end, ΔCAM values of stimulated pairs were compared to non‐stimulated control pairs recorded from the same network and time window. All controls were conditioned on the same presynaptic spike times as the stimulated pair, ensuring that no statistical biases arose from differences in spike counts (see Section 4.11 for details). The squared robust Mahalanobis distance ($D^{2}$) was used to quantify the multivariate separation of the stimulated pair from the control distribution in the ΔCAM plane. Assuming multivariate normality, $D^{2}$ was tested against a $\chi_{2}^{2}$ distribution (with two degrees of freedom) to assess the likelihood that the stimulated pair belonged to the control group. We then reported the associated probability, $P(\chi^{2}>D^{2})$, as the metric of deviation (see Section 4.12).

**FIGURE 4:**
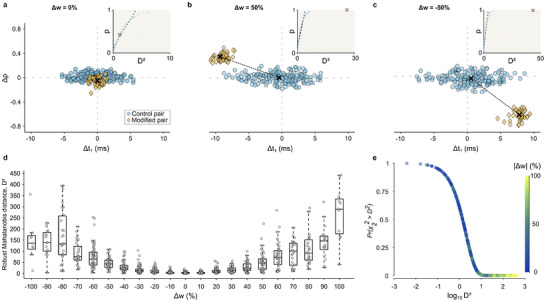
Statistical inference of synaptic weight changes using Conditional Activity Metrics (CAM) in in silico spiking neural networks. (a) CAM changes computed for a selected neuron pair (yellow diamonds) and multiple control pairs (blue circles) following no synaptic modification. For the selected pair, CAM was repeatedly computed (each yellow diamond) by resampling the presynaptic spike train (see Section S1.1) to obtain an unbiased estimate of the mean CAM change (black cross in yellow cluster). No statistical change was observed relative to the control cluster ($\chi_{2}^{2}$ test, $D^{2}=1.13$, $n_{controls}=185$, P=0.569; inset: $\chi_{2}^{2}$ empirical cumulative distribution function of squared robust Mahalanobis distances). (b) A 50% synaptic weight increase led to decreased spike latency and increased spike density. The yellow cluster shifted to the second quadrant of the ΔCAM plane and was identified as a statistical outlier ($\chi_{2}^{2}$ test, $D^{2}=25.92$, $n_{controls}=181$, P<0.001). (c) A 50% weight decrease produced the opposite effect, with the yellow centroid moving to the fourth quadrant and again identified as an outlier ($\chi_{2}^{2}$ test, $D^{2}=43.64$, $n_{controls}=183$, P<0.001). (d) Squared robust Mahalanobis distances increased systematically with larger synaptic weight changes across simulations. (e) The corresponding Chi‐square cumulative probabilities dropped sharply when weight changes exceeded ≈20–30%. Colors indicate the magnitude of applied synaptic change.

We illustrate the validity of the described analysis pipeline for three representative pairs drawn from in silico spiking neural networks. When no weight change was applied, the stimulated pair was indistinguishable from controls ($\chi_{2}^{2}$ test, $D^{2}=1.13$, $n_{controls}=185$, P=0.569; Figure 4a). Potentiation shifted ΔCAM toward shorter latency and higher density and was identified as a statistical outlier ($\chi_{2}^{2}$ test, $D^{2}=25.92$, $n_{controls}=181$, P<0.001; Figure 4b). In contrast, depression led to significant shifts in the opposite direction ($\chi_{2}^{2}$ test, $D^{2}=43.64$, $n_{controls}=183$, P<0.001; Figure 4c). Overall, weight changes could be inferred in 69.8% of the pairs based on a $\chi_{2}^{2}$ test of the squared robust Mahalanobis distance (349/500 neuron pairs, P<0.05; Figure S4a), with stronger effects near the extremes of the main diagonal (second and fourth quadrants; Figure S4d,g).

This trend was preserved across multiple in silico pairs and is summarized in Figure S4a(n=500 neuron pairs). Across simulations, detection sensitivity generally scaled with the magnitude of synaptic modification, with reliable separation emerging for changes exceeding ≈20–30% (Figure 4d,e).

In summary, CAM provides a scalable, statistically grounded method to infer network shaping, applicable to both single‐unit activity – spikes attributed to putative individual neurons after spike sorting – and multi‐unit activity (MUA) – spikes recorded at an electrode that potentially originate from more than one neuron. For our experimental data, we chose to compute CAM on MUA at the level of electrodes, since electrical stimulation was also delivered at this level. Consequently, our CAM readouts captured effective plastic changes in local subnetworks rather than in isolated single units. In the following sections, we experimentally demonstrate the utility of our platform with several complementary in vitro and *ex vivo* preparations. We utilized 2D cultures for their experimental ease and suitability for long‐term access, acute brain slices to confirm findings in preparations preserving native microcircuitry, and stem‐cell‐derived cerebral organoids as a physiologically‐relevant 3D model system.

### 2.4 Modulation of Synaptic Efficacy in Cultured Neuronal Networks

Cultures of primary dissociated rat cortical neurons offer stable experimental access for long durations and enable investigations into fundamental principles of neural computation [38, 39]. When maintained in vitro, these neurons self‐organize into large recurrent networks that preserve essential features of neuronal circuitry – stable yet plastic connections, spontaneous and evoked activity dynamics, and the capacity for adaptive reconfiguration [22, 40, 41, 42].

To demonstrate the efficacy of our HD‐MEA‐based circuit shaping pipeline, we first applied our platform to cultured neuronal networks between days in vitro (DIV) 11to31 and evaluated stimulation‐induced changes in local activity patterns. Our experimental design allowed us to induce and quantify both potentiation and depression of synaptic connections in the model system.

**FIGURE 5:**
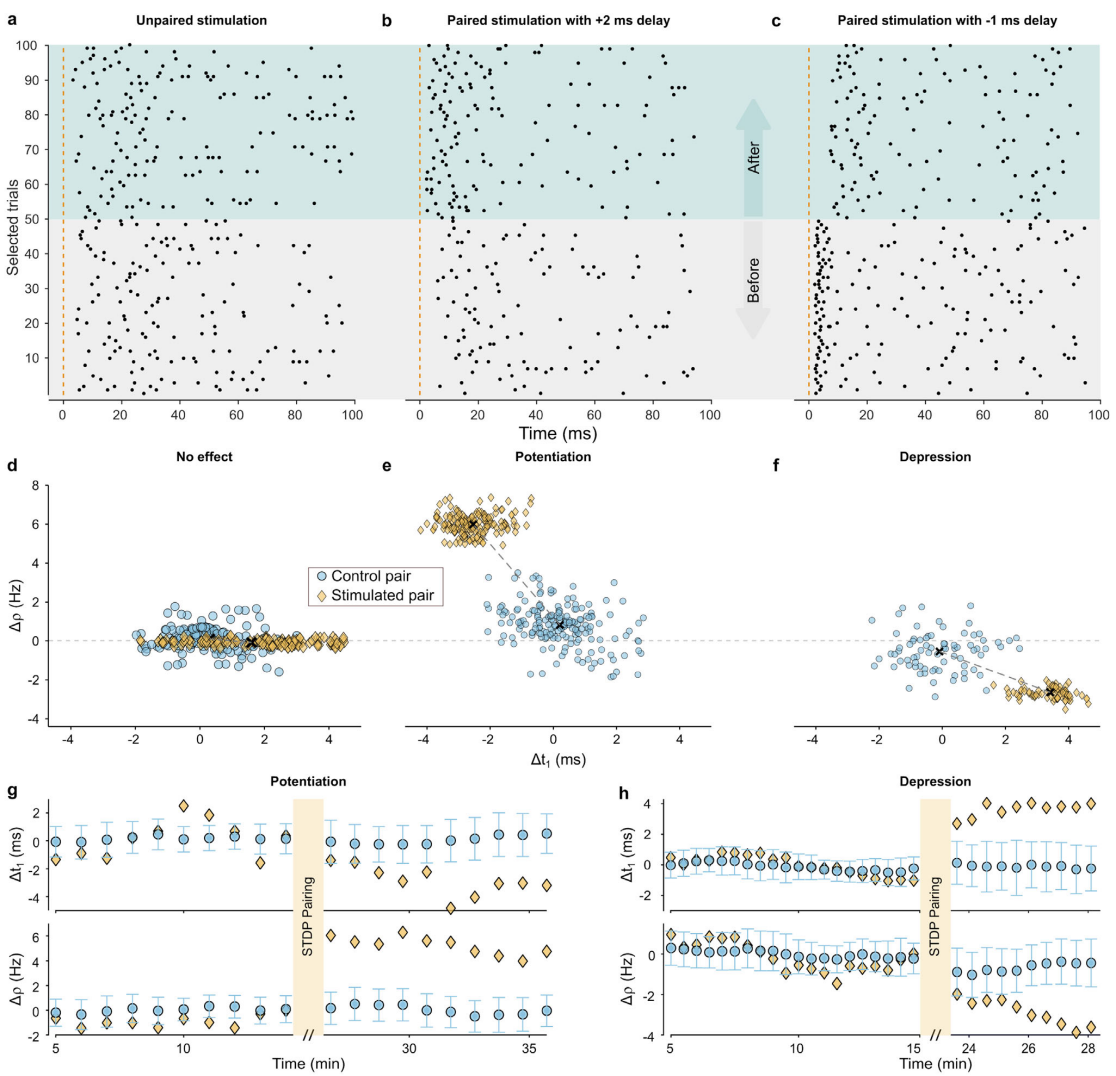
Application of the circuit shaping pipeline to dissociated rat cortical cultures on an HD‐MEA. (a–c) Representative spiking windows at the postsynaptic site, aligned with selected spikes at the presynaptic site before (gray) and after (teal) paired stimulation. (a) Unpaired control: stimulation was delivered only to the postsynaptic site and showed no changes in postsynaptic spiking. (b) +2 ms pairing resulted in reduced first‐spike latencies. (c) –1 ms pairing resulted in increased first‐spike latencies. (d–f) Conditional Activity Metrics (CAM) changes for stimulated pre–post pairs (yellow diamonds) vs. controls (blue circles). For the selected pair, presynaptic spikes were resampled to obtain an unbiased estimate of the CAM change (see Section S1.1). (d) Unpaired control: no effect ($\chi_{2}^{2}$ test, $D^{2}=3.35$, $n_{controls}=141$, P=0.19). (e) +2 ms pairing: ΔCAM shifted toward shorter latencies and higher spike densities ($\chi_{2}^{2}$ test, $D^{2}=23.14$, $n_{controls}=177$, P<0.001) indicative of local potentiation. (f) –1 ms pairing: ΔCAM shifted toward longer latencies and lower spike densities ($\chi_{2}^{2}$ test, $D^{2}=16.25$, $n_{controls}=78$, P<0.001), indicative of local depression). (g–h) Temporal evolution of ΔCAM (sliding 5 min windows) for the same pairs and networks in **b** and **c**. The shaded yellow region marks the recording interruption and re‐initialization period for the subsequent recording, in addition to the paired stimulation period. (g) For potentiation, 100 paired stimuli were delivered over 2 min after 15 min of baseline activity. Mean Δρ increased immediately and remained elevated (yellow diamonds), while mean $\Delta t_{1}$ showed a delayed but sustained decrease (yellow diamonds). (h) For depression, a similar stimulation protocol, but with a negative delay, was applied. Mean Δρ decreased and remained persistently suppressed (yellow diamonds), while mean $\Delta t_{1}$ initially increased before stabilizing at a higher value (yellow diamonds) relative to baseline and control pairs. In both cases, control pairs remained relatively unperturbed (blue circles and bars denote means and standard deviations).

Three different stimulation protocols were tested in the example networks shown in Figure 5: (1) unpaired control stimulation delivered only to the postsynaptic site, (2) paired stimulation with a +2 ms presynaptic‐to‐postsynaptic delay to induce potentiation, and (3) paired stimulation with a –1 ms delay (postsynaptic‐before‐presynaptic) to induce depression.

Representative examples of postsynaptic spiking windows aligned with presynaptic spikes revealed clear differences between conditions (Figure 5a–c). In the unpaired control case (Figure 5a), no changes in postsynaptic first spike latencies were observed following stimulation. In the causal pairing protocol (+2 ms; Figure 5b), there was a reduction in the latency to the first postsynaptic spike, indicative of strengthened synaptic connections. In contrast, anti‐causal pairing (–1 ms) increased this latency, suggesting a weakening of synaptic efficacy (Figure 5c).

These changes were quantified using ΔCAM. We resampled presynaptic spike trains with replacement (individual yellow diamonds in Figure 5d–f) to mitigate statistical bias in computing central estimates of ΔCAM at the stimulated pair (black cross in yellow clusters in Figure 5d–f; see also Section S1.1). Subsequently, the deviation of the mean $\Delta {\text{CAM}}_{\text{stim}}$ from the cluster of ΔCAM values of control pairs within the same network was used as the indicator of induced plasticity. In the unpaired control condition (Figure 5d), the centroid of the selected pair overlapped with the control cluster, confirming the absence of selective local changes ($\chi_{2}^{2}$ test, $D^{2}=3.35$, $n_{controls}=141$, P=0.19). However, following the +2 ms causal pairing (Figure 5e), the stimulated pair showed a clear shift toward shorter latencies and higher spike densities, emerging as a significant outlier indicative of localized potentiation ($\chi_{2}^{2}$ test, $D^{2}=23.14$, $n_{controls}=177$, P<0.001). Similarly, the –1 ms anti‐causal pairing (Figure 5f) produced a shift toward longer latencies and lower spike densities, which was also detected as a significant outlier, consistent with local depression ($\chi_{2}^{2}$ test, $D^{2}=16.25$, $n_{controls}=78$, P<0.001).

The temporal dynamics of these pairing‐induced changes were tracked using 5 min sliding windows (Figure 5g,h). For the causal pairing, we observed an immediate increase in Δρ following the stimulation period, while $\Delta t_{1}$ showed a delayed but sustained reduction (Figure 5g; 1 min increments). In the anti‐causal pairing protocol, Δρ decreased persistently after stimulation; $\Delta t_{1}$ initially increased before stabilizing at a higher value (Figure 5h; 30 s increments). Throughout both protocols, control pairs remained relatively unperturbed, confirming the specificity of the induced changes to the targeted connections (see also Section S1.1, Figure S2a).

In a subset of experiments, as described in Section 2.1, we tested the pipeline in high‐throughput mode, in which paired stimulation was delivered concurrently to multiple electrode pairs distributed across the array. Up to eight sites were stimulated in parallel on the same chip. The pairing delay was fixed at +3 ms, and the protocol was repeated 200 times at 1 Hz. From 89 electrode pairs stimulated in the high‐throughput mode, 41.5% (37/89) exhibited significant shifts in ΔCAM (P<0.05). These results demonstrate that the use of the platform can be scaled from single targeted pairs to the simultaneous manipulation of multiple distributed connections while preserving the capacity to induce robust and measurable plasticity.

Pooled results from multiple electrode pairs (n=227) are summarized in Figure S4b,e,h. Overall, strong evidence of plasticity induction was detected in 38.3% of pairs (87/227, P<0.05), with the largest effects clustering generally in the second and fourth quadrants of the ΔCAM plane. Crucially, control experiments confirmed that the changes in ΔCAM were specific to our paired stimulation protocol (see Section 4.10 and Section S1.2), as only 1 out of 36 tested site pairs exhibited a significant change in ΔCAM across all control conditions.

Anti‐causal pairing generally resulted in positive values of $\Delta t_{1}$ and negative values of Δρ. However, flipped effects were observed in many cases, particularly with causal pairing where positive STDP delays were applied (Figure S4e,h). In many cases, paired stimulation failed to produce ΔCAM shifts beyond control levels. Importantly, the pooled results include all tested pairs, without excluding cases where stimulation may have failed to reliably evoke direct neuronal responses. These response failures, which impede the expression of STDP, were confirmed during post‐hoc analysis in selected failed pairs (see Figure S9 for a representative example).

### 2.5 Circuit Shaping in Three‐Dimensional Neural Tissue

We next examined whether the HD‐MEA circuit‐shaping pipeline could be generalized to 3D neural tissues. Two complementary models were tested: acute mouse cortical slices and stem cell–derived brain organoids.

**FIGURE 6:**
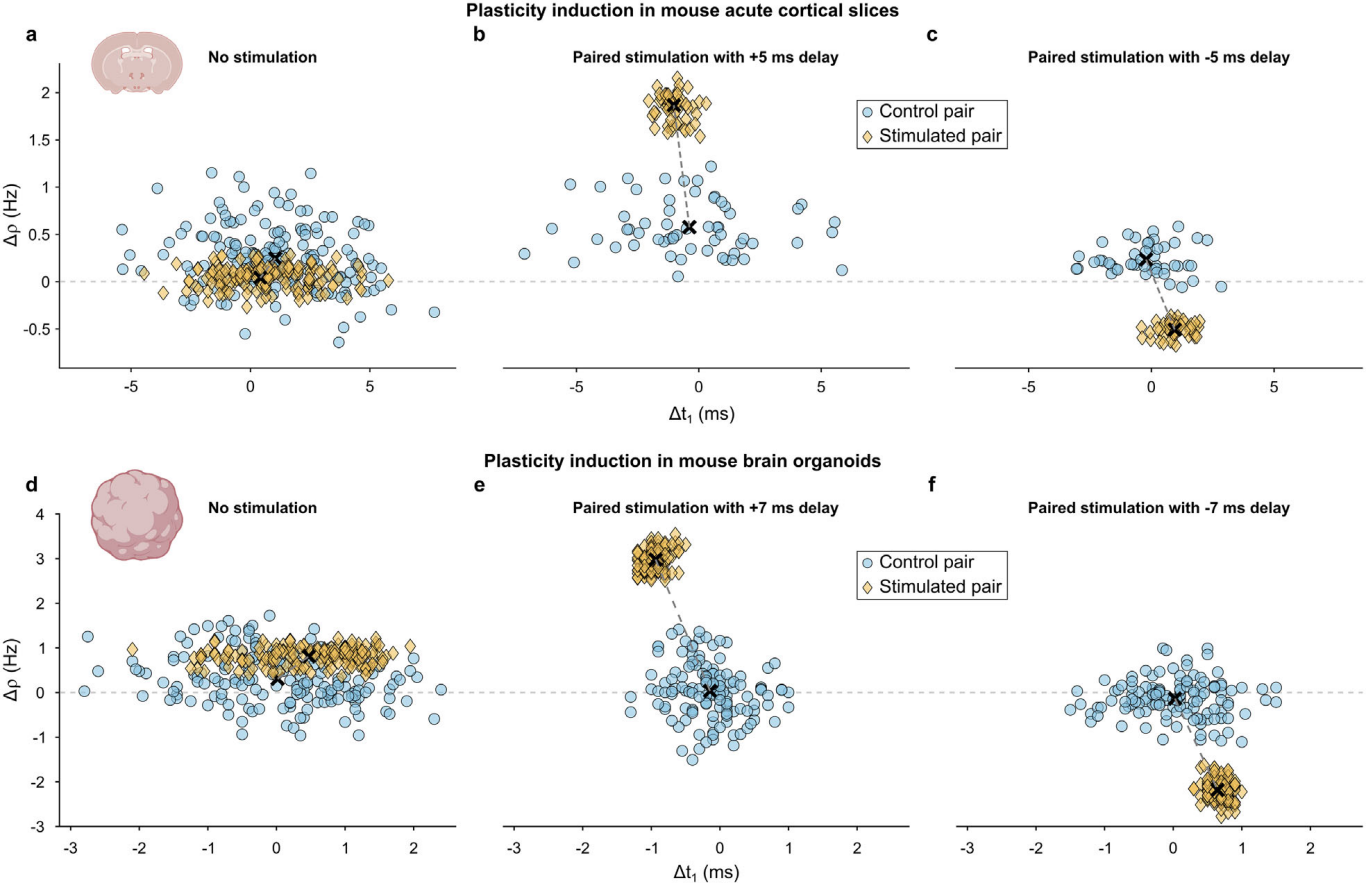
Extension of the circuit shaping pipeline to 3D tissue models. (a–c) Conditional Activity Metrics (CAM) changes in acute mouse cortical slices. Stimulated pre‐ and postsynaptic sites (yellow diamonds) are compared to control pairs (blue circles) from the same network as in Figure 5d–f. (a) Unstimulated control pair showed no significant effect ($\chi_{2}^{2}$ test, $D^{2}=0.46$, $n_{controls}=168$, P=0.79). (b) +5 ms pairing induced a significant ΔCAM shift toward higher spike densities ($\chi_{2}^{2}$ test, $D^{2}=21.67$, $n_{controls}=59$, P<0.001), indicating local potentiation. (c) ‐5 ms pairing produces a ΔCAM shift toward slightly longer latencies and lower spike densities ($\chi_{2}^{2}$ test, $D^{2}=26.20$, $n_{controls}=48$, P<0.001), indicating local depression. (d–f) CAM changes in cultured mouse brain organoids; the same comparison was made between stimulated pairs (yellow diamonds) and control pairs (blue circles). (d) Unstimulated control pair showed no significant effect ($\chi_{2}^{2}$ test, $D^{2}=0.53$, $n_{controls}=170$, P=0.53). (e) +7 ms pairing resulted in a pronounced ΔCAM shift toward higher spike densities and shorter first‐spike latencies ($\chi_{2}^{2}$ test, $D^{2}=17.37$, $n_{controls}=131$, P<0.001), indicating local potentiation. (f) –7 ms pairing shows a ΔCAM shift toward longer latencies and lower spike densities ($\chi_{2}^{2}$ test, $D^{2}=31.19$, $n_{controls}=122$, P<0.001), indicating local depression.

Acute slices preserve native cortical architecture, including laminar and columnar organization, and retain in vivo‐like synaptic motifs and excitatory‐inhibitory diversity in their native spatial arrangement, so that they provide a physiologically realistic substrate for probing local circuits. Their viability, however, is generally limited to a few hours, and only neurons in direct contact with the electrode plane can be monitored or stimulated. Acute slices (postnatal days 11–14) were placed on the HD‐MEA and stabilized with a custom anchor to ensure reliable electrical coupling (see Section 4.2). Our platform induced bidirectional changes in CAM: unstimulated control pairs remained stable ($\chi_{2}^{2}$ test, $D^{2}=0.46$, $n_{controls}=168$, P=0.79), while +5 ms pairing produced higher spike densities ($\chi_{2}^{2}$ test, $D^{2}=21.67$, $n_{controls}=59$, P<0.001), and –5 ms pairing produced the opposite effect: longer latencies and reduced densities ($\chi_{2}^{2}$ test, $D^{2}=26.20$, $n_{controls}=48$, P<0.001; Figure 6a–c).

Stem‐cell‐derived brain organoids recapitulate structural and functional hallmarks of early brain development, including neurogenesis, the emergence of excitatory–inhibitory balance, and spontaneous network dynamics [43, 44]. Their long‐term viability and scalability enable systematic studies of network maturation [45], plasticity [46], and disease‐relevant mechanisms [47, 48]. Despite incomplete maturation and structural variability, organoids are a powerful model for investigating how connectivity and information processing emerge during development. Applying our platform to organoids, cultured on HD‐MEAs, yielded qualitatively similar results: controls (unstimulated) remained stable ($\chi_{2}^{2}$ test, $D^{2}=0.53$, $n_{controls}=170$, P=0.53), while +7 ms pairing increased spike densities with modest latency reductions ($\chi_{2}^{2}$ test, $D^{2}=17.37$, $n_{controls}=131$, P<0.001), and ‐7 ms pairing decreased densities with slightly prolonged latencies ($\chi_{2}^{2}$ test, $D^{2}=31.19$, $n_{controls}=122$, P<0.001; Figure 6d–f; see also Section S1.1, Figure S2b).

Across both 3D models, plasticity signatures were dominated by changes in spike density (Δρ), with latency shifts being less pronounced than in dissociated cultures (Figure 5e–f). This difference likely reflects the more complex spatial organization and synaptic integration inherent in 3D architectures. Pooled results across *ex vivo* electrode pairs (n=52) confirmed robust plasticity induction in 38.5% of cases (20/52; P<0.05; Figure S4c). Together, these findings demonstrate that our platform induces selective strengthening or weakening of synaptic connections across diverse tissue architectures.

## 3 Discussion

We present a hardware‐enabled platform that combines CMOS HD‐MEAs with scalable analytics to selectively strengthen or weaken synaptic interactions across diverse in vitro and ex vivo systems, establishing a generalizable framework for evoking and verifying network plasticity. The platform's key feature is the parallel, low‐latency delivery of versatile stimulation protocols using state‐of‐the‐art HD‐MEAs. We demonstrate that targeted paired stimulation patterns can reliably evoke spatially confined neuronal responses and selectively modulate neuronal connection strengths.

We introduce CAM as a statistically rigorous quantitative parameter for characterizing induced plasticity. Conventional functional connectivity metrics (e.g., STP [31], STTC [32], DDC [49]) provide point estimates and lack measures of variability, limiting the reliability of comparisons across activity epochs. In contrast, CAM combines trial‐by‐trial measurements of postsynaptic first‐spike latencies and spike densities following individual presynaptic spikes – quantities known to be sensitive to changes in PSP strength  [35, 36, 37] – and summarizes them using robust, unbiased central‐tendency estimates. When conditioned on a sufficient number of presynaptic spikes, changes in CAM across epochs enable reliable comparisons between stimulated and unstimulated neuron pairs within the same network. This enabled us not only to infer subtle but systematic PSP strength changes – such as those observed in our patch‐clamp experiments (Figure 2) – but also to contextualize these changes relative to longitudinal controls.

CAM also enables temporal monitoring of relative changes in synaptic strength using a sliding window approach. Following an intervention, induced CAM changes were typically monitored for 20 to 30 min for stability. In selected pairs, where longer observation windows were used, effects were found to persist for at least 90min in both 2D cultures and cerebral organoids (Figure S2). Crucially, the delivery of repeated interventions using opposing protocols to the same pair resulted in a reversal of the induced modifications (Figure S3). These exploratory results establish the feasibility of using sequential interventions in realizing adaptive and closed‐loop scenarios.

Despite its advantages, CAM has limitations. Reliable estimations require prolific spiking of the target pair. Since subtle synaptic modifications may not be reflected as changes in spiking patterns, CAM may suffer from a sensitivity threshold. Further, CAM sensitivity is dependent on the temporal order of presynaptic contributions to cooperative postsynaptic firing (Figure S5), and network dynamics could further confound interpretation. Addressing these issues will require methodological advances.

Experimental results pooled across pairs and model systems revealed marked heterogeneity, likely depending on both experimental and biological factors. Reduced stimulation efficacy (e.g., due to low excitability  [30] or overlap with ongoing network activity  [50, 51]), faulty identification of preexisting connections, or short pairing durations may impede plasticity induction. Variability may also reflect the multifactorial nature of STDP, whose expression depends on neuron type, brain region, and spiking patterns  [12, 18, 52, 53, 54]. Furthermore, factors such as culture age, cell composition, and the presence of astrocytes may significantly influence the circuit's receptivity to plasticity, potentially limiting the universal effectiveness of a single induction protocol. In light of the richness of plasticity mechanisms operating in neuronal circuits, adaptive, empirical strategies that tune stimulation parameters in real‐time to achieve specific synaptic or circuit‐level modifications  [55] represent a compelling way forward. Our framework provides the necessary basis for such extensions.

Our platform offers a path toward next‐generation therapeutic neuromodulation strategies. Existing approaches directly drive or suppress action potential firing via electrical  [56, 57], chemogenetic  [58], or optogenetic  [59] means, often inducing uncontrolled plasticity in the network. Guided synaptic reorganization offers a more stable and efficient strategy  [8, 9]. Such targeted reshaping could ultimately inform therapies for spinal cord injuries  [60], neurotraumas  [61], and neurodegenerative disorders, such as Alzheimer's and Parkinson's disease  [62, 63].

The ability to induce and verify user‐defined connectivity patterns in neuronal ensembles represents a decisive step toward utilizing biological substrates for computation. Our platform provides promising opportunities in the field of wetware computing, where directed plasticity could help implement functional primitives, such as ensemble‐level logic operations  [64], which form the basis of scalable biohybrid architectures  [10, 65, 66].

Finally, our approach augments the experimental toolkit for basic neuroscience research. Unlike traditional stimulation paradigms, our system combines spatial precision with longitudinal analytical feedback, enabling controlled perturbation–response experiments at the synaptic level  [67, 68]. It will permit systematic tests of hypotheses about key concepts, including the role of leader neurons in ensembles  [69, 70], emergent synchrony  [71], and the interplay between intrinsic and synaptic homeostasis  [72]. Our pipeline is fundamentally modular, allowing individual components – from stimulation protocols to analytical routines – to be replaced or enhanced iteratively. This modularity makes the approach versatile and extensible to future applications in more complex in vivo systems [73, 74]. By bridging experimental precision with analytical rigor, our platform lays the foundation for advancements in neuroscience, neurotherapeutics, and biohybrid computing.

## 4 Experimental Section

### 4.1 Rat Primary Dissociated Cortical Cultures

The experimental protocols involving animals were approved by the Basel‐Stadt veterinary office in accordance with Swiss federal laws on animal welfare and were carried out according to the approved guidelines.

Pregnant Wistar rats (purchased from Janvier Labs, Le Genest‐Saint‐Isle, France) were anesthetized with 5% isoflurane and decapitated. Embryos (E18, both sexes) were immediately decapitated, and their brains were extracted and kept in ice‐cold HBSS (Thermo Fisher Scientific, 14025092) for the manual dissection of the cortices. Cortices were incubated in 0.25% Trypsin‐EDTA (Thermo Fisher Scientific, 25200‐056) at 37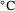 for 20 min with gentle swirling every 5 min to ensure uniform trypsin distribution. Tissue was then washed twice with warm Neurobasal cell medium (Thermo Fisher Scientific, 21103‐049; 37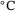).

Cortices were mechanically triturated and diluted in Neurobasal cell medium to 1000 cells/μL. A 36 μL cell suspension (≈36000 cells) was seeded onto HD‐MEA chips pre‐sterilized in 70% ethanol (30 min), rinsed thrice with sterile water, and coated overnight with poly‐D‐lysine (PDL; Sigma–Aldrich, P6407) at 37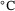 and 5% CO_2_. Excess PDL was removed, and the chip was washed three times with sterile water immediately before plating.

Neurons were allowed to settle for 45 min in the incubator (37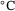 and 5% CO_2_) before adding 2 mL plating medium, composed of Neurobasal (Thermo Fisher Scientific, 21103‐049), 10% Horse Serum (Thermo Fisher Scientific, 26050088), 0.5 mM GlutaMAX (Thermo Fisher Scientific, 35050‐038), and 2% B‐27 Plus (Thermo Fisher Scientific, A3582801). Half of the medium was exchanged with maintenance medium (Brainphys (Stemcell Technologies, 05790), 2% NeuroCult SM1 (Stemcell Technologies, 05711), and 0.2% Penicillin‐Streptomycin (10,000 U/mL) (Thermo Fisher Scientific, 15140122)) twice a week. Cultures were maintained at 5% ${\mathrm{CO}}_{2}$, 37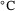, and 90% humidity.

Our study relies on 25 neuronal networks derived from 8 different pregnant rats, yielding 227 electrode pairs and an additional 36 pairs for control experiments. Recordings spanned days in vitro (DIV) 11–31.

### 4.2 Acute Mouse Cortical Brain Slices

Mouse pups (P12‐14; both sexes; C57BL/6J purchased from the Jackson Laboratory, Maine, USA, and locally bred) were decapitated under deep isoflurane anesthesia, followed by brain dissection in ice‐cold artificial cerebro spinal fluid (aCSF) bubbled with carbogen gas (95% $O_{2}$, 5% ${\mathrm{CO}}_{2}$). The following aCSF recipe was used (in mM): 126 NaCl, 3.5 KCl, 1.25 ${\mathrm{NaH}}_{2}\mathrm{PO}$ 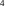, 1 ${\mathrm{MgSO}}_{4}$, 1.2 ${\mathrm{CaCl}}_{2}$, 26 ${\mathrm{NaHCO}}_{3}$, and 10 glucose, at approximately pH 7.3 when bubbled with carbogen. Perfusion with aCSF using such a recipe resulted in naturalistic cortical Up‐Down state oscillations; UP states manifested at a relatively low frequency, so that little interference with plasticity induction protocols occurred. Coronal brain slices 370μm) were prepared using a vibratome (VT1200S, Leica, Wetzlar, Germany) with a sterile razor blade. Under a stereomicroscope, the slices were further trimmed to isolate the somatosensory cortex, which matched the electrode area of our HD‐MEA in size. Slices were stored in a submerged chamber in carbogenated aCSF at room temperature until use. For plasticity experiments, brain slices were transferred to the sensing area of the CMOS HD‐MEA, fixed with a custom‐designed slice anchor, and perfused with heated aCSF (32 to 34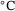 throughout the experiment). Paired stimulation was delivered to 19 electrode pairs with slices obtained from 6 mice.

### 4.3 Cultured Mouse Cerebral Organoids

Mouse‐derived cerebral organoids (mCOs) were generated from the commercially available CGR8 embryonic stem cell (ESC) line, which is derived from the 129S2/SvPasOrlRj mouse strain (Institute of Human Biology, Roche). Organoids were transferred to our laboratory at day in vitro (DIV) 9 and maintained in 10 mm diameter petri dishes in neural maturation medium composed of (1:1) DMEM‐F12/HEPES: Neurobasal, 0.5× N2 supplement, 1× B27 (+Vitamin A), 5 μg/ml insulin, 0.4 mM ascorbic acid, 1 mg/ml ${\mathrm{NaHCO}}_{3}$, 1× GlutaMAX, 0.5× MEM‐NEAA, 1× penicillin‐streptomycin, and 0.1 mM 2‐mercaptoethanol. Petri dishes were placed on an orbital shaker (INFORS HT, Bottmingen, Switzerland) at 37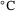 in 5% ${\mathrm{CO}}_{2}$, with half‐medium changes performed every 2to3 days. At DIV 18, the mCOs were washed in ice‐cold HBSS containing ${\mathrm{Ca}}^{2+}$ and ${\mathrm{Mg}}^{2+}$ (ThermoFisher, 14025092), embedded in low‐melting agarose 3% (wt/vol) (Sigma–Aldrich, A9414), and kept on ice for 20 min until ready for slicing. Organoids were sectioned into (300 μm) slices in ice‐cold HBSS with ${\mathrm{Ca}}^{2+}$ and ${\mathrm{Mg}}^{2+}$ using a vibratome (VT1000S, Leica, Wetzlar, Germany) with a sterile razor blade and collected in cell culture inserts (Merck Millipore, PICM0RG50) in a six‐well tissue culture dish. Slices were cultured at the air–liquid interface (ALI) to improve oxygen and nutrient supply, as described by Giandomenico et al. [75] for at least 24 h. Before plating the slices onto the high‐density microelectrode arrays (Maxtwo 6‐well plate, MaxWell Biosystems, Zurich, Switzerland), each chip was coated with 0.05% polyethylenimine (PEI; Sigma–Aldrich, 408727) prepared in borate buffer (pH 8.5; Thermo Scientific, 28341) and incubated at 37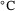 with 5% ${\mathrm{CO}}_{2}$. After a 1 h incubation, the chips were rinsed three times with distilled water and allowed to dry. To promote tissue adhesion, the chips were then coated with laminin (0.02 mg/ml; Sigma–Aldrich, 67407300) and incubated overnight at 37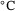 with 5% ${\mathrm{CO}}_{2}$. Media changes were performed every 2to3 days. Electrophysiological recordings and stimulation protocols were performed at DIV 28 and DIV 34. The analysis included 33 electrode pairs from 5 cerebral organoids.

### 4.4 In Silico Spiking Neural Networks

We generated ground‐truth datasets using spiking neural network (SNN) simulations implemented in Brian2 (version 2.8.0)  [76]. Each network consisted of 2000 Hodgkin–Huxley‐type neurons (1600 excitatory, 400 inhibitory) connected via conductance‐based (COBA) synapses  [77] with fixed connection probabilities: 5% for excitatory and 20% for inhibitory neurons. Synaptic delays were drawn from a squared‐uniform distribution, favoring shorter delays and promoting local connectivity. Networks exhibited spontaneous firing and irregular bursting, driven by noisy external currents injected into all neurons.

To model targeted plasticity, we selectively manipulated synaptic strengths between selected neuron pairs. COBA synapses were modulated using a parameter referred to as the synaptic weight, w. Simulations proceeded in two stages. First, we ranked neurons by mean firing rates in an initial run. One pair was randomly selected from the top decile. The synaptic weights of connected neurons were drawn uniformly from [0.1,0.9], except for the selected pair, whose weight was varied systematically.

In the second stage, 33 simulations were performed, varying the synaptic weight $w_{sel}$ of the selected pair from 0 to 1 in 0.1 increments across three synaptic delays ($t_{sd}=1,\;5,\;\text{and}\;10\mathrm{ms}$). All other weights and synaptic delays remained fixed. No other activity‐dependent plasticity mechanisms were implemented to ensure that changes in network dynamics were attributable solely to the imposed parameter changes.

To evaluate the causal effect of Δw on CAM values, we compared the first half of a simulation with parameters $\left(w_{\text{sel}},\;t_{\text{sd}}\right)$ to the second half of a different simulation with $\left(w_{\text{sel}}+\Delta w,\;t_{\text{sd}}\right)$. This halving‐and‐stitching procedure simulated targeted changes in $w_{\text{sel}}$ while controlling for other dynamics. The protocol yielded 363 distinct Δw samples, forming a centered triangular distribution spanning the range [−1,1] per selected pair. To enhance robustness and generalizability, the entire procedure was repeated across 5 networks (10 pairs in total) initialized with different random seeds.

### 4.5 High‐Density Microelectrode Array Recordings

A CMOS HD‐MEA system was used to record extracellular signals from the different in vitro and *ex vivo* models. The HD‐MEA featured 26,400 electrodes covering a sensing area of approximately $3.85\times 2.10{\mathrm{mm}}^{2}$, and the center‐to‐center electrode distance (pitch) was 17.5μm. Up to 1024 electrodes could be arbitrarily connected to the read‐out channels to simultaneously record from neurons at 20 kHz sampling frequency [23].

Cultured mouse brain organoids were recorded using the six‐well commercial variant of the chip (MaxTwo) from MaxWell Biosystems (Zurich, Switzerland) at 10 kHz sampling frequency. For primary dissociated cultures and acute brain slices, we used the original chips with custom in‐house post‐fabrication packaging steps briefly described below.

Each chip was wire‐bonded to a custom‐made printed circuit board. A plastic ring was glued to the chip surface to help contain the encapsulation material. Either a small plastic frame or a dam out of highly viscous epoxy was also added around the electrode array to prevent subsequent epoxy from covering it. Epoxy was then applied using a syringe to protect the bonding wires, with care taken to avoid covering the electrode array.

A thin layer of platinum (Pt‐black) was subsequently electrodeposited on the electrodes to increase the effective electrode‐neuron interfacing area and reduce the electrical impedance of the electrodes. The uniformity of the deposition was verified optically under a microscope.

The reference electrode is integrated on chip and has been fabricated simultaneously with the microelectrode array in platinum. It consists of eight platinum bars (two on each side of the electrode array), providing a total surface area of approximately 0.55 ${\text{mm}}^{2}$. The reference electrodes, like the working electrodes, are coated with Pt‐black.

### 4.6 Electrical Stimulation

Using a matrix of switches placed below the electrodes, the HD‐MEA system allows the user to connect an arbitrary selection of electrodes to 32 voltage stimulation units located outside the electrode area. The stimulation circuits are grouped into two blocks, each comprising 16 units and three 10‐bit digital‐to‐analog converters (DACs). By selecting different DAC outputs, complex stimulation patterns can be delivered to each stimulated electrode.

For our experiments, positive‐first biphasic pulses were delivered, with each phase of the pulse lasting 200 μs. For ramp protocols, to test stimulation efficacy, amplitudes ranging from 50 to 500 mV were explored. Based on an assessment of stimulation efficacy (see Figure 2c for illustration) amplitudes ranging from 250 to 400 mV were selected for STDP experiments. It should be noted that the stimulation efficacy illustrated in Figure 2c may not directly reflect the outcome for every targeted site. In particular, the efficacy depended on the morphological features of the cellular compartments located near the selected stimulation sites, as well as on sample‐specific factors. For selected control cases, a 0 mV sham stimulation was also delivered (see also Section 4.10).

For paired stimulation protocols, the presynaptic component consisted of a single biphasic pulse. The postsynaptic component was a burst of five to ten pulses at 50 to 200 Hz. The individual pulse parameters were always symmetric between pre‐ and postsynaptic components. This stimulation pattern was repeated 100to200 times at intervals of 1 s.

Temporal delays between the pre‐ and post‐stimulation components, ranging from −10to+10ms, were implemented to bias synaptic modification toward potentiation or depression. Negative paired delays were implemented by flipping the pre‐ and postsynaptic sites (see also Section 4.10).

### 4.7 Simultaneous Whole‐Cell Patch‐Clamp and HD‐MEA Experiments

To validate our circuit shaping strategies, we used the patch‐clamp technique to measure the cellular voltage responses evoked during a typical HD‐MEA‐induced plasticity experiment. We employed a patch‐clamp setup with an integrated and post‐hoc synchronized CMOS HD‐MEA system, as previously described  [78]. Primary rat neuronal cultures (DIV 8‐21) plated on HD‐MEA chips were transferred to the combined setup and perfused with Brainphys media (Stem Cell Technologies, 05790) at physiological temperature.

Borosilicate glass micropipettes (OD: 1.5 mm, ID: 0.86 mm; Science Products, BD150‐86‐10) were pulled and fire‐polished (DMZ Universal Electrode Puller, Zeitz Instrumente Vertriebs GmbH) yielding pipette resistances of 5 to 6 MΩ. The micropipettes were backloaded with the following internal solution (concentration in mM): 120 K‐Gluconate (Sigma–Aldrich, G4500), 6 KCl (Sigma–Aldrich, P3911), 4 NaCl (Sigma–Aldrich, S9888), 10 HEPES (Sigma–Aldrich, H3375), 0.2 EGTA (Sigma–Aldrich, E0396), 0.3 Tris‐GTP (Sigma–Aldrich, G9002), 2 Mg‐ATP (Sigma–Aldrich, A9187), and 10 Glucose (Sigma–Aldrich, G8270); the pH was adjusted with KOH (Sigma–Aldrich, 484016) to 7.35, and the osmolarity was adjusted to 290 mOsm/L with sucrose (Sigma–Aldrich, S7903). For visualization of the cell morphology, a small amount of Alexa Fluor 488 Hydrazide (Thermo Fisher Scientific, A10436; 2 mM dissolved in $H_{2}$0) was added to the internal solution on the recording day to reach a final concentration of 20 μM.

Patch‐clamp data in current‐clamp mode were sampled at 20 kHz and low‐pass filtered at 10 kHz, with data acquisition controlled by pClamp (Clampex 11.4, Molecular Devices) and Multiclamp Commander (version 2.2.2, Molecular Devices).

Upon establishing the whole‐cell current‐clamp mode, the cell was first localized on the HD‐MEA chip. For this localization, spikes were induced in the patched cell by applying brief current‐injection pulses. HD‐MEA electrodes exhibiting correlated spiking activity were considered to be near the soma of the target cell. To assess the reliability of extracellular action potential induction in the patched cell, we applied our standard biphasic voltage stimulus to different HD‐MEA electrodes near the cell soma (at 1 Hz). Stimulation of the electrodes in the direct vicinity of the cell soma typically induced action potentials with high reliability for the defined stimulus parameters.

To probe for synaptic inputs, we first performed a brief assessment of spontaneous network spiking activity. We then stimulated putative presynaptic neurons, choosing the electrode that featured the largest extracellular signal amplitude as the stimulation site. A putative excitatory monosynaptic connection was identified when a probing stimulus (at 1 Hz) at the presynaptic site exhibited a correlated excitatory postsynaptic potential in the patched cell. The identified pre‐ and postsynaptic stimulation sites were then used for the subsequent spike timing‐dependent plasticity (STDP) experiment.

### 4.8 Data Acquisition

For data acquisition, HD‐MEAs were interfaced with a dedicated recording setup that supports bidirectional communication between the chip and the host computer. Recordings and stimulation protocols were managed with the software MaxLab Live (MaxWell Biosystems, version 23.2.2), which provides both a graphical user interface (GUI) and a Python API. Chips were acclimated for ≈20 min prior to data collection. Activity scans and network recordings were performed using either the GUI or the API, while stimulation protocols were implemented via the API. All data were stored in the Hierarchical Data Format (HDF5).

A schematic overview of the experimental pipeline is depicted in Figure 1g. Before initiating data acquisition, an activity scan was performed to optimize the electrode configuration. The entire sensing area of the chip was divided into seven regions, each recorded for 120 to 300 s. This preliminary scan of spontaneous activity identified the most active regions, facilitating the design of a spatial electrode configuration that concentrated around the most responsive electrodes, indicating putative neural units. The spike detection threshold was chosen in the MaxLab Live software as 5 times the root mean squared amplitude of the signal at each electrode. In most cases, we used clusters of 20 electrodes selected around the responsive sites, ensuring a minimum inter‐cluster spacing of 100 µm, resulting in ≈1000 electrodes per configuration. This electrode configuration was subsequently used to perform a 15 to 45 min baseline spontaneous activity recording to identify target electrode pairs for subsequent stimulation protocols, as described in Section 4.9. These target pairs were subjected to the stimulation patterns detailed in Section 4.6.

Following the selection of candidate electrode pairs, recordings were performed either with the same configuration or with a denser configuration of electrodes around the selected sites. The higher density enabled more precise targeting of stimulation to the peak‐amplitude regions of each neuron's electrical footprint and provided redundancy for artifact‐cleaning procedures when assessing stimulation efficacy. If a different electrode configuration was chosen, a further 15 min baseline spontaneous recording was performed, after which the stimulation protocol was applied. Following stimulation, the system was allowed to stabilize for 1 min before a subsequent 15 to 20 min spontaneous recording session. This sequence of before‐ and after‐stimulation recordings enabled comparative analyzes of neuronal activity using CAM. The stimulation and post‐recording steps were iteratively repeated with different stimulation protocols or electrode pairs.

### 4.9 Pair Selection

Putative directly connected multi‐unit pairs were identified through a cross‐correlation analysis pipeline applied to spike recordings from the HD‐MEA. First, electrodes were selected based on predefined amplitude and firing rate criteria. Specifically, electrodes with peak amplitudes and firing rates above the 25^th^ percentile across the array were retained. From this subset, pairwise cross‐correlograms (CCGs) were computed using 0.4 ms bins and a symmetric ±150 bin window, yielding a set of cross‐correlation matrices for all retained electrode pairs.

For each pair (i,j), we computed the spike transmission probability (STP;  [31]),

$${\mathrm{STP}}_{i,j}=\frac{1}{N_{i}}\sum_{k\in W}\left({\mathrm{CCG}}_{i,j}\left(k\right)-{\hat{B}}_{i,j}\left(k\right)\right)$$

where $N_{i}$ is the number of presynaptic spikes, W is a post‐zero‐lag time window (0.4–8 ms), and ${\hat{B}}_{i,j}\left(k\right)$ is the baseline CCG estimated via convolution with a hollow Gaussian kernel. Statistical significance of transmission was assessed by computing P‐values under a Poisson model, comparing observed CCG counts to ${\hat{B}}_{i,j}$. Pairs with any significant bins (P<0.01) were flagged as potentially connected.

Candidate connections were retained if they exceeded a minimum transmission threshold ($|{\mathrm{STP}}_{i,j}|>0.03$) and fell within a biologically plausible inter‐electrode distance range (50–500 μm). Additional quality control criteria were applied to filter spurious correlations: (i) pairs were excluded if fewer than 30% of bins in the CCG contained non‐zero counts, and (ii) overly synchronized pairs were removed based on heuristic criteria, including outlier peak magnitudes (>8× mean, >5× std) or excessively sharp peaks near zero lag.

Remaining candidate pairs were deduplicated and sorted by absolute STP value. The final set was partitioned into tertiles – low, medium, and high transmission – based on empirical STP quantiles. These categories were used in downstream analyzes to stratify pairwise interactions by transmission strength.

Subsequently, a visual curation step was performed to further refine pair selection. Raw signal cutouts around spike events were made on each candidate electrode, typically drawn from the medium and high tertile ranges, and average extracellular footprints were visually inspected to confirm distinct spatial profiles. If necessary, electrode selections were adjusted to align with local minima within each footprint. This refinement was guided by prior work showing that electrical stimulation at sites of maximal extracellular signal amplitude was more likely to elicit action potentials in response to electrical stimulation  [29].

### 4.10 Paired Stimulation

The target electrodes corresponding to the positions of the identified putative neuron pairs on the HD‐MEA were stored and transferred into custom written Python scripts. The number of electrodes targeted simultaneously was user‐defined and could be customized to align with the specific experimental requirements.

During the paired stimulation protocol, the presynaptic site received a single pulse, followed by a chosen delay of Δt ms, after which the postsynaptic site was stimulated with a burst of pulses (see Section 4.6). For negative delays, the pre‐ and postsynaptic sites were internally flipped in the script. In these cases, the original presynaptic site would receive a burst of pulses Δt ms after a single pulse was delivered to the postsynaptic site.

The delay parameter Δt between pre‐ and postsynaptic sites was selected based on the desired experimental outcome. Three distinct stimulation protocols were employed:

- *Single Pair Targeted Stimulation*: A single pair of electrodes was stimulated using a delay value determined by the initial spike transmission probability assessment. In general, shorter absolute delays Δt were employed to induce stronger synaptic changes.
- *Multiple Pairs with Uniform Delay*: Multiple electrode pairs were simultaneously stimulated using the same delay Δt, with the number of repetitions increased to 250 to ensure robust synaptic modification.
- *Systematic Delay Variation for a Single Pair*: A single pair was stimulated and recorded in six iterations, for example, with delays of −6,−4,−2,+2,+4,+6 ms (not necessarily in sequential order, nor those specific values).

These protocols provide flexibility for exploring STDP mechanisms and enabled precise manipulation of temporal delays. To rigorously assess whether the observed changes in synaptic strength were attributable specifically to the stimulation pattern employed – and not to non‐specific stimulation effects – we implemented a series of control conditions administered either before or after the standard paired stimulation protocols on selected pairs:

- *Random Delay Stimulation*: Selected pairs of electrodes were stimulated using a delay Δt ms randomly selected at each iteration (out of 100 total), with values randomly drawn from symmetric intervals (e.g., −50ms, +50ms). This protocol was designed to determine whether CAM modifications were specifically dependent on precise spike timing – as expected following STDP rules – or whether they were merely the result of stimulating the pre‐ and postsynaptic sites.
- *Random Electrode Stimulation*: In this control experiment, a pair of electrodes was randomly selected from a dense configuration (electrode pitch = 17.5μm), explicitly avoiding spatial overlap with the footprints of the electrode pair designated by the main experimental pipeline. This surrogate pair was stimulated using the same timing parameters as those applied to the original pair, either preceding or following the main pairing protocol. This condition was intended to discern whether CAM changes were spatially specific to targeted neuronal pairs or reflective of more global network perturbations.
- *Zero‐Amplitude Stimulation*: The standard stimulation protocol was applied to the original electrode pair, but the stimulation amplitude was set to zero. This control was introduced to rule out the potential influence of the routing of the stimulation circuits on subsequent spontaneous recordings, independent of actual current injection.
- *Pre‐Only and Post‐Only Stimulation*: In this unpaired control condition, the stimulation protocol was selectively applied to either the pre‐ or postsynaptic site. The complementary site was left unstimulated. This condition served to assess the impact of incomplete stimulation (e.g., presynaptic failure) on the protocol's effectiveness and the resulting CAM dynamics.

### 4.11 Selection of Controls

Control electrode pairs were selected to match the baseline activity profile of the stimulated pair while preserving the same presynaptic site. This design decision was motivated by the properties of the Conditional Activity Metric (CAM), which estimates per‐pair directional spike timing and conditional spike density based on reference spike‐triggered windows (see Section 2.3, Section S1.1). Since CAM values depend critically on the temporal structure and number of spikes from the reference (presynaptic) spike train, preserving the presynaptic site across all controls ensures both CAM stability and consistency of estimation biases across stimulated and control pairs.

Two CAM objects were instantiated – one for pre‐stimulation and one for post‐stimulation epochs – using extracellular spike recordings. The CAM vector for a pair (i,j) was defined as:

$${\text{CAM}}_{control}^{i,j}=\left(\begin{array}{c} t_{1}^{i,j}\\ \rho^{i,j} \end{array}\right)$$

where $t_{1}^{i,j}$ is the median first‐spike latency in j following spikes in i, and $\rho^{i,j}$ is the mean spike density in j within a predefined window following each spike in i.

These pseudo‐pairs (i.e., they may or may not be functionally connected) were formed by pairing the stimulated presynaptic electrode $e_{\text{pre}}$ with unstimulated postsynaptic electrodes across the array. To avoid contamination from the stimulation protocol, electrodes were excluded from control candidacy if they (i) participated directly in stimulation (either as a post‐electrode or co‐activated pre‐electrode) or (ii) were located within 200 μm of either stimulation site. The latter criterion was iteratively relaxed if sufficient control electrodes were unavailable. A pre‐screening step further excluded electrodes with low spike counts (below 25% of the stimulation‐site average) to ensure CAM estimates would converge reliably.

CAM metrics were computed for all control pseudo‐pairs, both before and after stimulation. The change in CAM, denoted as:

$$\Delta {\text{CAM}}_{control}^{i,j}=\left(\begin{array}{c} \Delta t_{1}^{i,j}\\ \Delta \rho^{i,j} \end{array}\right)$$

was used as a surrogate for stimulation‐induced synaptic modulation. Pairs with undefined or extreme values in either component (quartile outliers) were excluded.

The final control set thus consisted of pseudo‐pairs (i.e., not necessarily connected) with shared presynaptic input and no exposure to stimulation. This design ensured that ΔCAM differences reflected the selective impact of stimulation on postsynaptic partners, while minimizing confounds due to estimation variability or presynaptic sampling bias.

We also accounted for the possibility that electrical stimulation at the target sites could trigger the co‐activation of distal neurons via axons of passage, potentially biasing the statistical assessment of plasticity effects at the target pair. In the event that such antidromically activated neurons were functionally connected – either to each other or to the target pair – they could, in theory, exhibit similar plasticity phenotypes. However, by maintaining a broad selection of control pairs for statistical comparison, we ensured that any potential ‘stray’ activation effects remained non‐systematic and did not bias the statistical evaluation of changes at the target pairs.

### 4.12 Statistical Detection of Stimulation‐Induced Outliers

To assess whether the stimulated pair exhibited a statistically significant change in CAM relative to the control distribution, we computed robust Mahalanobis distances in the ΔCAM space. Let $C={\left\{\Delta {\text{CAM}}_{k}^{i,j}\right\}}_{k=1}^{N}$ be the set of ΔCAM vectors for N control pairs. We estimated the robust mean μ and covariance matrix Σ of the control distribution using the minimum covariance determinant (MCD) estimator  [79]. The squared Mahalanobis distance for the stimulated pair's mean ΔCAM, ${\text{CAM}}_{\text{stim}}$, was then computed as:

$$D^{2}={\left({\text{CAM}}_{\text{stim}}-\mu \right)}^{⊤}\Sigma^{-1}\left({\text{CAM}}_{\text{stim}}-\mu \right).$$

Reliable estimation of ${\mathrm{CAM}}_{\text{stim}}$ requires prolific spiking of the target pair in both the before and after (post‐stimulation) periods. Consequently, pairs exhibiting sparse spiking activity (less than 25 spikes) in either measurement phase, after the proximity mask exclusion (see Section S1.1), were excluded from subsequent analysis.

Under the assumption that the control cluster follows a multivariate normal distribution, the squared Mahalanobis distances would theoretically follow a $\chi^{2}$ distribution with two degrees of freedom. $D^{2}$ was thus compared to the $\chi_{2}^{2}$ distribution. The null hypothesis was that stimulated pairs were drawn from the same multivariate distribution as the controls.

(2) $$P=1-F_{\chi_{2}^{2}}\left(D^{2}\right),$$

where $F_{\chi_{2}^{2}}$ is the cumulative distribution function of a chi‐squared distribution with two degrees of freedom. P, by definition, represents the probability of observing a larger squared Mahalanobis distance under a $\chi^{2}$ distribution with matching degrees of freedom. Small values of P indicate that the stimulated pair is an outlier relative to the distribution of matched control pairs.

Quantile–quantile (Q–Q) plots of $D^{2}$ values from control pairs were used to visualize the fit to the $\chi_{2}^{2}$ distribution and to highlight the position of the stimulated pair relative to the control population. This approach provides a multivariate, distribution‐aware test of stimulation specificity that accounts for the covariance between $\Delta t_{1}$ and Δρ across the control ensemble.

### 4.13 Pooled Visualization of Stimulated Pairs

For each stimulated pair, we computed its ΔCAM and normalized it relative to the mean of the corresponding control pairs:

$$\Delta {\text{CAM}}_{\text{norm}}=\Delta \text{CAM}-E\;\left[\Delta {\text{CAM}}_{\text{control}}\right].$$

To enable pooled visualization across heterogeneous scales, each component z of $\Delta {\text{CAM}}_{\text{norm}}$ was subsequently rescaled to the unit interval by

$$f\left(z\right)=\mathrm{sgn}\left(z\right)\;\left(1-e^{-E_{z}\left|z\right|}\right),$$

where $E_{z}>0$ is a scaling constant chosen to balance the dynamic range and prevent saturation at the plot boundaries.

Empirically selected parameters were:

- in silico: $E_{x}=0.1$, $E_{y}=1.2$ (larger $E_{y}$ due to smaller Δρ magnitudes);
- 2D in vitro: $E_{x}=E_{y}=0.2$ (similar magnitudes across dimensions);
- 3D *ex vivo*: $E_{x}=0.3$, $E_{y}=0.6$ (smaller $\Delta t_{1}$ relative to Δρ).

The pooled normalized results across model systems are summarized in Figure S4.

### 4.14 Artifact Removal and Trace Cleaning

To assess the direct responses evoked by electrical stimulation, we implemented an artifact cleaning procedure for raw microelectrode array recordings based on hybrid local regression. The method combined nonlinear least‐squares optimization for accurate modeling of artifact morphology with polynomial regression for the efficient correction of later regions. This approach preserves signal integrity while minimizing non‐physiological distortions.

The raw voltage traces $X\in R^{T\times C}$, where T denotes the samples and C the channels, were aligned to the stimulation indices ${\left\{t_{\text{stim}}^{i}\right\}}_{i=1}^{N}$. Baseline noise statistics were estimated from a 1 s pre‐stimulation window. High‐pass filtering (>10 Hz) was applied, and the noise covariance matrix $\Sigma_{\text{noise}}$ was computed. Synthetic Gaussian noise samples, $Z\left(t\right)∼N\left(0,\Sigma_{\text{noise}}\right)$ were generated using Cholesky decomposition of $\Sigma_{\text{noise}}$ to preserve cross‐channel correlations and were later used to impute data in invalid segments.

For each stimulation time, $t_{\text{stim}}^{i}$, saturation (ADC clipping) and decay phases were identified using adaptive search procedures. Saturated segments and their immediate decays were flagged and excluded from model fitting. A pre‐stimulation window $W_{\text{pre}}=\left[t_{\text{stim}}^{i}+\Delta_{1},\;t_{\text{stim}}^{i}+\Delta_{2}\right]$ was used to estimate the baseline offset δ to ensure continuity with the preceding activity.

Artifact‐contaminated regions were corrected in two stages:

1. *Initial decay (nonlinear fit)*. The steep artifact onset was modeled using a constrained exponential decay function
$$f_{\exp}\left(t;a,b,c\right)=a\;e^{-bt}+c,$$
  fit using nonlinear least‐squares with an interior‐point algorithm. The constraints enforce continuity at the stitch point $\left(t_{0},x_{0}\right)$ nearest to the artifact boundary.
2. *Later correction (linear fit)*. Subsequent regions were modeled with a degree‐3 polynomial
$$f_{\text{poly3}}\left(t\right)=\alpha_{3}t^{3}+\alpha_{2}t^{2}+\alpha_{1}t+\alpha_{0},$$
  fit via ordinary least‐squares. This step reduced computational costs while adequately correcting residual baseline shifts.

The fit quality was assessed by comparing residual errors against noise‐scaled thresholds:

$$\frac{1}{M}\sum_{j=1}^{M}{\left(f\left(t_{j}\right)-x\left(t_{j}\right)\right)}^{2}<\varepsilon \sigma^{2},$$

where σ is the channel‐specific noise standard deviation, and M is the number of fitted points. M=15, and ε=1 were empirically chosen for our data. The cleaned trace was then obtained by subtracting the fitted artifact model from the raw signal and restoring the local baseline offset:

$$x_{clean}\left(t\right)=x\left(t\right)-f\left(t\right)+\delta ,$$

where δ is the pre‐stimulus offset estimated from $W_{\text{pre}}$.

Any intervals marked as invalid (for example, during saturation or poor fits) were replaced with Gaussian noise sampled from $N(0,\Sigma_{\text{noise}})$ and adjusted by the local mean offset, δ. This procedure maintained the per‐channel spectral and cross‐channel covariance structures of the raw signal.

The cleaning algorithm was parallelized across channels to scale to HD‐MEA channel counts. The cleaned signals, $X_{\text{clean}}$, were subsequently bandpass filtered 250 to 3000 Hz, IIR) to extract spiking activity.

### 4.15 Statistical Analysis

To test if ΔCAM values of the stimulated pairs were outliers relative to their corresponding control cluster, we used the chi‐square test of the squared robust Mahalanobis distance with two degrees of freedom, as described in Section 4.12.

Pairwise associations between ΔCAM components and imposed synaptic weight changes for in silico neuron pairs were assessed using the Pearson product–moment correlation coefficient,

$$r=\frac{\sum_{i}\left(x_{i}-\bar{x}\right)\left(y_{i}-\bar{y}\right)}{\sqrt{\sum_{i}{\left(x_{i}-\bar{x}\right)}^{2}\sum_{i}{\left(y_{i}-\bar{y}\right)}^{2}}},$$

with significance evaluated against the null hypothesis of zero correlation using the corresponding t‐distribution.

Changes in EPSP amplitudes before and after the pairing protocol were assessed using a left‐tailed two‐sample t‐test, with the null hypothesis that the mean post‐pairing amplitude was equal to the mean pre‐pairing amplitude.

All analyzes were performed in MATLAB (versions 23.2–25.1; The MathWorks, Natick, MA, USA).

## Author Contributions

F.C., J.B., S.K., and A.H. jointly conceived and supervised the project. R.S., F.C., J.B., and S.K. planned and designed the study. R.S., F.C., and S.K. implemented the technical framework. S.K., R.S., and Y.B. designed and implemented the analytical pipeline. S.K., Y.B., R.S., J.B., and T.G. acquired the raw data. J.‐S.D. developed the mouse brain organoid protocol and cultured organoids to day 9. J.‐S.D., L.S., M.S., and J.G.C. provided neural organoid expertise. J.G.C. oversaw the planning and logistics of organoid technology transfer. S.K., Y.B., and J.B. analyzed the data and interpreted the results with inputs from F.C. and A.H. S.K., Y.B., and J.B. prepared the figures. S.K. and Y.B. wrote the original draft with inputs from J.B., T.G., L.S., M.S., and A.H. All authors contributed to manuscript revisions and approved the final version.

## Ethics Statement

Our methods were carried out in accordance with the guidelines approved by the Basel–Stadt veterinary office according to Swiss federal laws on animal welfare.

## Conflicts of Interest

J.‐S.D. and J.G.C. are current employees of F. Hoffmann‐La Roche Ltd. The company provided support in the form of salaries for the authors but did not have any additional role in the study design, data collection and analysis, decision to publish, or preparation of the manuscript.

The other authors declare no competing interests.

## Supporting information


**Supporting File**: advs75473 sup 0001 SuppMat.pdf.

## Data Availability

The data that support the findings of this study are available from the corresponding author upon reasonable request.
