# Supplementary material for: Engineering Neuronal Network Connectivity Through Precise and Scalable Electrical Modulation
Source: Adv Sci (Weinh). 2026 May 7;13(43):e75473. doi: 10.1002/advs.75473 (PMC13335577; doi:10.1002/advs.75473)
Supplement: Supplementary file 1 — Supporting File: advs75473 sup 0001 SuppMat.pdf. [file ADVS-13-e75473-s001.pdf]

Engineering Neuronal Network Connectivity through Precise and Scalable Electrical Modulation

Sreedhar S. Kumar, Yannaël Bossard, Rachel Sava, Tobias Gänswein, Lorenca Sadiraj, Jean-Samuel Dupré, Manuel Schröter, J. Gray Camp, Fernando Cardes, Julian Bartram, Andreas Hierlemann*

Dr. S. S. Kumar, Y. Bossard, R. Sava, T. Gänswein, L. Sadiraj, Dr. M. Schröter, Dr. F. Cardes, Dr. J. Bartram, Prof. A. Hierlemann
Bio Engineering Laboratory, Department of Biosystems Science and Engineering, ETH Zurich, Klingelbergstrasse 48, CH-4056, Basel, Switzerland.
Email Address: sreedhar.kumar@bsse.ethz.ch
Y. Bossard
Université Paris-Saclay, Ecole Normale Supérieure Paris-Saclay, 4 Av. des Sciences, 91190, Gif-sur-Yvette, France.
J.-S. Dupré, Prof. J. G. Camp,
Institute of Human Biology (IHB), Roche Pharma Research and Early Development, Roche Innovation Center Basel, CH-4051, Basel, Switzerland.
J.-S. Dupré
Quantitative Developmental Biology Laboratory, Department of Biosystems Science and Engineering, ETH Zurich, Klingelbergstrasse 48, CH-4056, Basel, Switzerland.
Prof. J. G. Camp,
Biozentrum, University of Basel, Spitalstrasse 41, CH-4056, Basel, Switzerland.

S.K., F.C., J.B., and A.H. contributed equally as senior authors.

S1 Supporting Information

S1.1 Conditional activity metrics (CAM)

Conditional Activity Metrics (CAM) quantify the relative spike timing and conditional spike density between pairs of spike trains. Formally, let $S_i = \{s_k\}_{k=1}^{N_i}$ and $S_j = \{t_\ell\}_{\ell=1}^{N_j}$ denote the spike times of neurons at electrodes i and j, respectively. Consider a fixed first-spike search window $W_{t_1} = [\tau_{\min}^{t_1}, \tau_{\max}^{t_1}]$ and a density search window $W_\rho = [\tau_{min}^{\rho}, \tau_{max}^{\rho}]$, both relative to each reference spike. Then, for each reference spike $s_k \in S_i$, we define the conditional random variables:

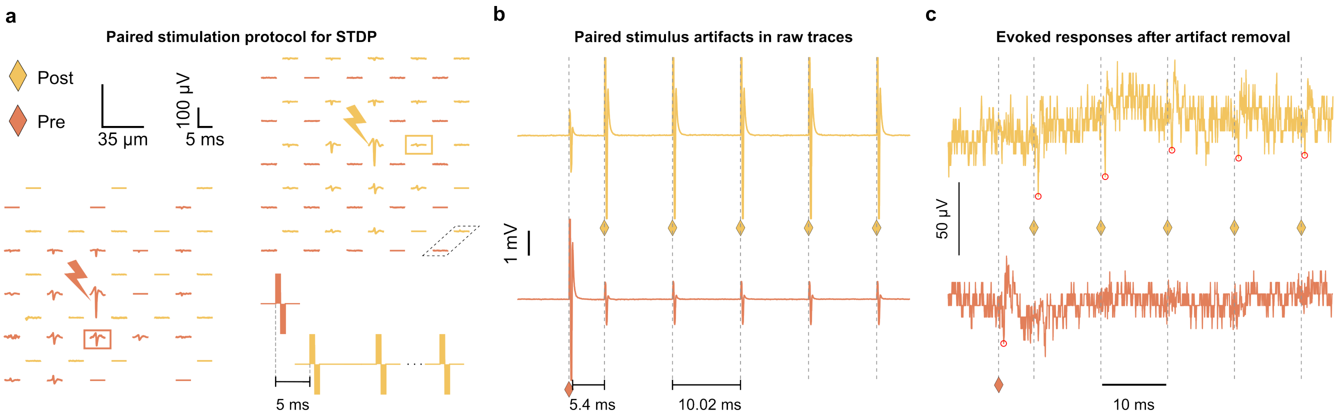

Figure S1: **Validation of temporal precision of paired stimulation (a)** The dual-site paired stimulation protocol included a single presynaptic pulse, followed 5 ms later by a 5-pulse 100 Hz postsynaptic train. Footprints (average extracellular electrical potentials of neurons across array electrodes during action potentials) were computed at routed electrodes during spontaneous activity before paired stimulation. The electrodes featuring the highest-amplitude signals in each footprint were selected as stimulation sites (yellow: postsynaptic; red: presynaptic). Footprints were shifted diagonally to avoid overlap and aid visualization (black parallelogram indicates overlapping signal snippets). **(b)** Raw traces from electrodes adjacent to stimulation sites (colored boxes in **d**; presynaptic: red; postsynaptic: yellow; diamonds: stimulation times) during paired stimulation. Artifacts confirm sub-millisecond timing precision (pre-post delay: 5.4 ms; inter-pulse interval: 10.02 ms). **(c)** Artifact-suppressed traces from the same electrodes as in **e**, showing spatially confined, time-locked responses (red circles: direct responses).

$$T_1^{i,j}(s_k) = \min\left\{\, t_\ell - s_k \;:\; t_\ell \in S_j,\; t_\ell - s_k \in W_{t_1} \,\right\}, \tag{S1}$$

$$\rho^{i,j}(s_k) = \frac{1}{|W_\rho|}\,\Big|\,\left\{\, t_\ell \in S_j \;:\; t_\ell - s_k \in W_\rho \,\right\}\Big|, \tag{S2}$$

where $|W_\rho|$ denotes the interval length, and $|\{\cdot\}|$ denotes the cardinality of the set. If no $t_\ell \in S_j$ falls in $s_k + W_{t_1}$, then $T_1^{i,j}(s_k)$ is considered censored (that is, treated as missing); in practice, we exclude censored events from the median estimate.

The CAM for the pair (i,j) is the vector-valued conditional random variable (conditioned on a presynaptic spike at time s_k):

$$\mathrm{CAM}^{i,j}(s_k) = \begin{pmatrix} T_1^{i,j}(s_k) \\ \rho^{i,j}(s_k) \end{pmatrix}.$$

To report a single descriptive CAM per electrode pair, we aggregate across all reference spikes s_k. Concretely, we define the summary statistics

$$t_1^{i,j} = \operatorname{median}\big(\, T_1^{i,j}(s_k) \;:\; T_1^{i,j}(s_k) \text{ observed} \,\big), \tag{S3}$$

$$\rho^{i,j} = \frac{1}{N_i}\sum_{k=1}^{N_i} \rho^{i,j}(s_k) \approx \mathbb{E}\big[\rho^{i,j}\big], \tag{S4}$$

where the median in Equation S3 is taken over those reference spikes for which a first postsynaptic spike falls within W_t (i.e., non-censored events). The sample mean in Equation S4 estimates the conditional expectation of spike density. The reported CAM vector is then

$$\mathrm{CAM}^{i,j} = \begin{pmatrix} t_1^{i,j} \\ \rho^{i,j} \end{pmatrix}$$

To minimize the overlap of postsynaptic windows in cases of bursting in the reference spike train, we optionally applied a proximity mask: a refractory dead time of 100 ms was imposed following each reference spike $s_k \in S_i$. Any subsequent reference spikes occurring within this interval were excluded from the conditional analysis. This ensured that only well-separated presynaptic events contributed to the estimation of $t_1^{i,j}$ and $\rho^{i,j}$. This procedure reduced redundancy and ensured a more reliable estimation of conditional activity metrics.

In *in silico* spiking neural networks, CAM components were strongly correlated with synaptic strengths, specifically for a network-embedded neuron pair with systematically varying synaptic weights (Figure S7a). Both t_1 and ρ showed statistically significant correlations with synaptic strengths ($\rho:\ n = 198,\ r = 0.77,\ P < 0.001;\ t_1:\ n = 198,\ r = -0.79,\ P < 0.001$).

However, CAM was not suitable for estimating absolute synaptic strengths between arbitrary neuron pairs, as the metric lacks a natural normalization that would allow for direct comparison across neuron pairs.

Hence, in our approach, the difference in CAM metrics of defined neuron/unit/electrode pairs between a window of activity before and after the intervention was used as a measure of the selective impact of the intervention. ΔCAM was defined as:

$$\Delta\mathrm{CAM}^{i,j} = \begin{pmatrix} \Delta t_1^{i,j} \\ \Delta\rho^{i,j} \end{pmatrix} \tag{S5}$$

ΔCAM exhibited a strong association with relative changes in synaptic strengths in *in silico* SNNs (Figure S7e).

Selection of temporal windows for CAM

The temporal windows W_{t_1} and W_ρ were empirically determined based on performance across multiple simulation runs. For all tested synaptic weight changes Δw, the timing metric Δt_1 showed the strongest deviation from the null distribution when using a window of $W_{t_1} = [1, 90]$ ms. Similarly, the density metric $\Delta\rho$ exhibited maximal sensitivity with $W_\rho = [1, 30]$ ms. These optimal ranges are tightly coupled to the kinetics of the postsynaptic potentials (PSPs) used in our models (Figure 1b), which are variable and generally inaccessible in extracellular recordings.

Both Δt_1 and $\Delta\rho$ demonstrated reduced trial-to-trial variability with increasing window length. However, this increase in stability came at the cost of reduced effect magnitude – particularly for $\Delta\rho$ – suggesting a trade-off between reliability and sensitivity. Based on these observations, we adopted $W_{t_1} = [2, 85]$ ms and $W_\rho = [5, 100]$ ms, relative to each spike in the reference train, as the default analysis windows for all subsequent CAM computations (Figure 1c); however, they were slightly adjusted during the analysis period.

Convergence and resampling of CAM estimates

Since CAM is defined as a conditional random variable, each estimate is derived from repeated conditional observations aligned with presynaptic events. For a given pair (i, j), the per-spike samples

$$t_1^k \sim T_1^{i,j}, \quad \rho^k \sim \rho^{i,j}$$

represent individual observations of the underlying conditional latency and density distributions. The aggregate estimates

$$\hat{t}_1^{i,j} = \text{median}\{t_1^k\}_{k=1}^N, \qquad \hat{\rho}^{i,j} = \frac{1}{N}\sum_{k=1}^{N} \rho^k$$

represent noisy realizations of the true CAM vector that converge to the underlying values as the number of reference spikes N increases.

To reduce statistical bias in estimating changes in CAM metrics (ΔCAM) from limited trial data, we employed a paired bootstrap resampling approach. For both pre-stimulation (before) and post-stimulation (after) conditions, we generated $B = 50$ bootstrap CAM replicates by randomly sampling with replacement to create subsets S^* containing 75% of the original presynaptic spikes from set S_i. This yielded resampled CAM estimates for each condition:

$$\widehat{\text{CAM}}_{b,\text{before}}^{i,j} = \begin{pmatrix} \hat{t}_1^{i,j}(S^*_{b,\text{before}}) \\ \hat{\rho}^{i,j}(S^*_{b,\text{before}}) \end{pmatrix}, \quad \widehat{\text{CAM}}_{b,\text{after}}^{i,j} = \begin{pmatrix} \hat{t}_1^{i,j}(S^*_{b,\text{after}}) \\ \hat{\rho}^{i,j}(S^*_{b,\text{after}}) \end{pmatrix}, \quad b = 1, \ldots, B$$

We then computed all possible ΔCAM differences between the pre- and post-stimulation bootstrap samples:

$$\Delta\text{CAM}_{b_1,b_2}^{i,j} = \widehat{\text{CAM}}_{b_2,\text{after}}^{i,j} - \widehat{\text{CAM}}_{b_1,\text{before}}^{i,j}, \quad b_1, b_2 = 1, \ldots, B$$

yielding $B^2 = 2500$ difference estimates. The centroid of this distribution of differences,

$$\overline{\Delta\text{CAM}}^{i,j} = \frac{1}{B^2}\sum_{b_1=1}^{B}\sum_{b_2=1}^{B} \Delta\text{CAM}_{b_1,b_2}^{i,j},$$

was used as our robust estimate of ΔCAM for the stimulated pair. Since this was a computationally

expensive procedure, we performed this bootstrap resampling step only for the stimulated pairs and not for the individual control pairs. This approach provides a more accurate characterization of the true change in conditional activity metrics by accounting for variability in both pre- and post-stimulation conditions, thereby minimizing estimation bias and enabling rigorous statistical comparisons against control pair distributions. Note that for visualizations, only a subset of the 2500 difference estimates, randomly selected to match in cardinality with the number of selected control pairs, was used (for example, yellow clusters in Figure 5d-f, Figure 6).

Empirically (based on *in silico* spike trains), we found that $\rho^{i,j}$ converged more rapidly than $t_1^{i,j}$, consistent with its larger integration window W_ρ and its reliance on multiple postsynaptic events. Convergence was defined as the point where estimates fell within 5% of the final value (computed from all reference spikes; Figure S5d). Across a range of synaptic weights, $\rho^{i,j}$ stabilized earlier than $t_1^{i,j}$ (Figure S5e), with both components typically converging using less than 70% of the total reference spikes.

Based on these findings, we fixed the resampling fraction at 75% for all stimulated pairs. The number of spikes required for reliable detection of ΔCAM was inversely related to the magnitude of synaptic modification $|\Delta w|$ – large changes were detectable with shorter recordings (Figure S5f). As the effect size could not be predicted *a priori*, we standardized recording durations to 15 to 20 min for pre- and post-stimulation across all experiments.

Long-term monitoring of CAM

To resolve the dynamics of plasticity induction, CAM was tracked over time using a sliding-window approach. For a given time t, the sliding-window formulation, CAM(t) is defined as:

$$\mathrm{CAM}^{i,j}(t) \;=\; \begin{pmatrix} t_1^{i,j}(t) \\ \rho^{i,j}(t) \end{pmatrix},$$

where

$$t_1^{i,j}(t) \;=\; \mathrm{median}\left\{T_{1,k}^{i,j} \;\middle|\; s_k \in S_i \cap W(t)\right\}, \quad \rho^{i,j}(t) \;=\; \mathbb{E}\left[\rho_k^{i,j} \;\middle|\; s_k \in S_i \cap W(t)\right].$$

Here, $W(t) = [t - \Delta, t)$ is a sliding analysis window of width $\Delta = 5\,\mathrm{min}$, $T_{1,k}^{i,j}$ is the first-spike latency of neuron j conditioned on a reference spike $s_k \in S_i$, and $\rho_k^{i,j}$ is the postsynaptic spike density within the analysis window following s_k. Each $\mathrm{CAM}^{i,j}(t)$ was normalized by subtracting the mean baseline CAM computed over the 20 min pre-stimulation period.

This procedure yields trajectories of $t_1^{i,j}(t)$ and $\rho^{i,j}(t)$, allowing direct visualization of the temporal course of synaptic modifications (Figure S2).

Representative examples in dissociated cultures and brain organoids show that stimulation often produced a rapid shift in CAM immediately following paired stimulation, which could manifest independently in latency (t_1) or density (ρ). In many cases, this initial change persisted for several minutes before partially returning toward baseline or stabilizing at an intermediate plateau. In some instances, the squared Mahalanobis distance continued to increase well beyond the stimulation period, indicating broader changes in network firing statistics.

The subsequent evolution was variable across preparations: in some cases, CAM stabilized near baseline, whereas in others, it fluctuated markedly, consistent with ongoing changes in the network states. Extended recording sessions were occasionally associated with a progressive reduction in overall spiking activity, which decreased the reliability of CAM estimates over long timescales.

S1.2 Additional control experiments

To confirm that the observed changes in CAM were specifically induced by our structured STDP stimulation protocols, rather than by nonspecific effects of electrical stimulation, we conducted a series of control experiments (see section 4).

Random Delay Stimulation: When delays were randomly selected for each instance of the stimulation, no significant changes in ΔCAM were observed across the 12 electrode pairs tested ($P > 0.05$ for all cases). Notably, 9 of these pairs exhibited significant ΔCAM shifts when tested with our standard paired stimulation protocol, either before or after the random-delay control condition.

Random Electrode Stimulation: When electrode pairs for stimulation were selected randomly, without applying the standard pair-selection procedure, no significant changes in ΔCAM were observed across the three tested pairs ($P > 0.05$). Notably, one of these pairs showed a significant ΔCAM change when subsequently tested with the standard paired stimulation protocol.

Zero-Amplitude Stimulation: When stimulation amplitudes were set to zero, no significant changes in ΔCAM were observed across the four electrode pairs tested ($P > 0.05$). Notably, three of these pairs showed significant ΔCAM shifts when subsequently tested with our standard paired-stimulation protocol.

Pre-Only and Post-Only Stimulation: Unpaired stimulation protocols were applied in either the pre-only or post-only mode across 17 electrode pairs. Postsynaptic-only stimulation did not produce significant changes in ΔCAM in any of the eight pairs tested ($P > 0.05$). Three of these pairs showed significant ΔCAM shifts when tested with the STDP-like paired-stimulation protocol, either before or after this unpaired control. During presynaptic-only stimulation, one of nine pairs exhibited a significant decrease in ΔCAM, consistent with long-term depression typically induced by low-frequency presynaptic activation [1]. None of these pairs were subsequently tested with the

STDP protocol.

S1.3 Systematic delay variation

To probe delay dependence more systematically, we applied sequential stimulation protocols with varying pre–post delays to single electrode pairs (section 4). Experiments were conducted on 9 pairs from rat primary cortical cultures and 2 pairs from mouse cerebral organoids. Each session began with a 10 min baseline recording, followed by six stimulation blocks interleaved with 10 min spontaneous activity. Delays were symmetric in the range -10 to $+10$ ms and presented in ascending or descending order (e.g., +2/–2, +4/–4, +6/–6 ms; or –7/+7, –5/+5, –3/+3 ms).

Responses were heterogeneous across pairs. In total, 81.8% (9/11) showed at least one significant ΔCAM shift ($P < 0.05$) after stimulation, consistent with either potentiation or depression. Four pairs exhibited two consecutive and opposing effects (i.e., the potentiation induced by a causal protocol was abolished by a subsequent anti-causal one), often occurring during the first or last two blocks. No pair showed more than two consecutive significant effects, suggesting that synaptic modifications may not track rapid alterations in imposed plasticity and instead may require a refractory period before becoming responsive to further adaptations.

S1.4 Further characterization of CAM

CAM components exhibited tight correlations with synaptic strengths in network-embedded neuron pairs (Figure S7a). A selected neuron pair was subjected to a systematic variation of synaptic weights. Both components, t_1 and ρ, expressed relative to their baseline values at null synaptic weight ($t_1 - t_1(w = 0)$ and $\rho - \rho(w = 0)$), were tightly correlated with synaptic strength (ρ: $r(196) = 0.77$, $P < 0.001$; t_1: $r(196) = -0.79$, $P < 0.001$).

In Figure S7b-c, we evaluated the influence of the temporal windows W_{t_1} and W_ρ across simulations. For all tested values of synaptic weight change Δw, Δt_1 exhibited maximal differentiation with the window $W_{t_1} = [1, 90]$ ms. Similarly, the peak of differentiation was obtained with $W_\rho = [1, 30]$ ms for $\Delta\rho$. These observations are intrinsically linked to the dynamics of PSPs, visible in Figure S5b, which are not directly accessible via extracellular recordings. Importantly, both $\Delta\rho$ and Δt_1 demonstrated stabilization of their values as the windows increased in duration. The stable values are at lower absolute magnitudes than the observed maximal differentiations, especially for $\Delta\rho$, implying that extended windows reduce the sensitivity of the ΔCAM to changes in Δw. While this attenuation may reduce the amplitude of differentiation, it offers the advantage of minimizing arbitrary or ad hoc selection of window parameters. Guided by these results, we selected $W_{t_1} = [1, 90]$ ms and $W_\rho = [1, 100]$ ms as the standard analysis windows for subsequent simulations.

Using the convergence criterion illustrated in Figure S7d, we quantified the proportion of the presynaptic neuron's spike train required for CAM convergence across different values of synaptic weights. As shown in Figure S7e, we observed that ρ typically converged faster than t_1, which is consistent with the broader integration window of W_ρ and its dependence on more postsynaptic spikes, thus enhancing robustness. On average, both ρ and t_1 required less than 70% of the presynaptic spike train for convergence. Based on these findings, we adopted a 75% resampling of presynaptic spike times to generate the distribution of ΔCAM values in subsequent analyses of the stimulated neuron pairs.

As one would expect, Figure S7f demonstrates that the magnitude of synaptic change $|\Delta w|$ inversely correlates with the number of spikes needed to detect a reliable change in ΔCAM—i.e., stronger changes required shorter recording durations. Nevertheless, because the effect size of STDP-like stimulation was typically not predictable in advance, the duration of pre- and post-stimulation recordings was fixed at 15 minutes for all experiments.

We conducted a systematic evaluation of ΔCAM relative to established metrics of synaptic efficacy, employing previous simulations to benchmark sensitivity to synaptic weight perturbations (Figure S8). Spike Transmission Probability (STP; [2]; 0.4 ms bins and post-zero-lag time window of 0.4–2.8 ms after subtracting the imposed synaptic delays) exhibited negligible correspondence with weight changes ($r_{\Delta \mathrm{STP}}(498) = -0.14$), consistent with its original purpose as a detector of strong monosynaptic interactions within narrowly defined temporal windows. Although significant improvements were observed when extending the detection window, the metric remained ill-suited for capturing graded changes in synaptic efficacy. In contrast, the Spike Time Tiling Coefficient (STTC; [3]; correlation time window of 20 ms) showed a robust positive correlation with weight changes ($r_{\Delta \mathrm{STTC}}(498) = 0.82$), reflecting its sensitivity to local synchrony at broader temporal scales, albeit with limited statistical tractability for resampling.

Dynamical Differential Covariance (DDC; [4]; 5 ms bins, $\sigma_{\mathrm{kernel}} = 3$ ms and a kernel cutoff set to $\lceil 5\sigma_{\mathrm{kernel}} \rceil$) also exhibited a substantial correlation with weight changes ($r_{\Delta \mathrm{DDC}}(498) = 0.64$), in line with its model-based assumptions that emphasize directional interactions but are vulnerable to mis-estimation of the time-derivative under non-smooth, sparse, and noisy spike trains. Notably, ΔCAM displayed the strongest alignment with weight-dependent changes ($r_{\Delta\rho}(498) = 0.93$; $r_{\Delta t_1}(498) = -0.91$), confirming its capacity to integrate the temporal precision of spike-conditioned analyses with the statistical robustness afforded by bin-free local neighborhood sampling. Collectively, these findings indicate that ΔCAM captures synaptic weight dependencies more faithfully than conventional approaches.

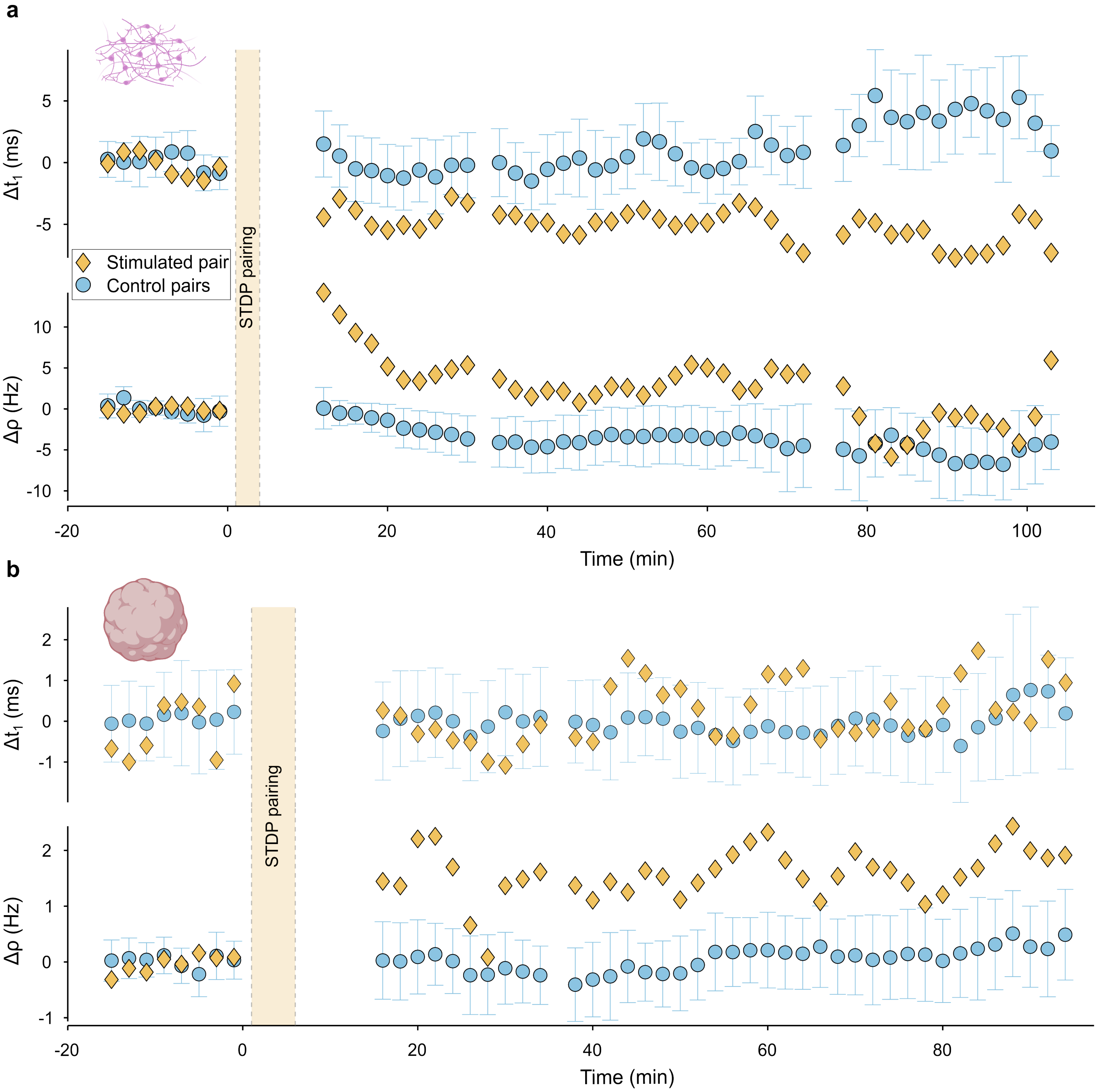

Figure S2: **Temporal tracking of Conditional Activity Metrics (CAM).** CAM features were computed using a 5 min sliding window with 2 min increments. Each data point was normalized by subtracting the mean CAM computed during the 20 min pre-stimulation baseline period. Yellow diamonds represent mean values at the stimulated pair, and blue circles and whiskers represent the mean (SD) of concurrently recorded unstimulated pairs drawn from the same network. **(a)** A representative example showing the sustained post-intervention changes in CAM features over a 105 min period for a stimulated electrode pair in rat primary dissociated cortical cultures. Notably, while the change in spike density ($\Delta\rho$; lower) returned toward baseline levels at around 80 min, the modification in the first spike latency (Δt_1; upper) persisted. **(b)** The same analysis was applied to a stimulated pair in a cultured mouse cerebral organoid sample, showing sustained changes over a 90 min window. In this case, the effect of the intervention was reflected predominantly in the sustained increase in spike density.

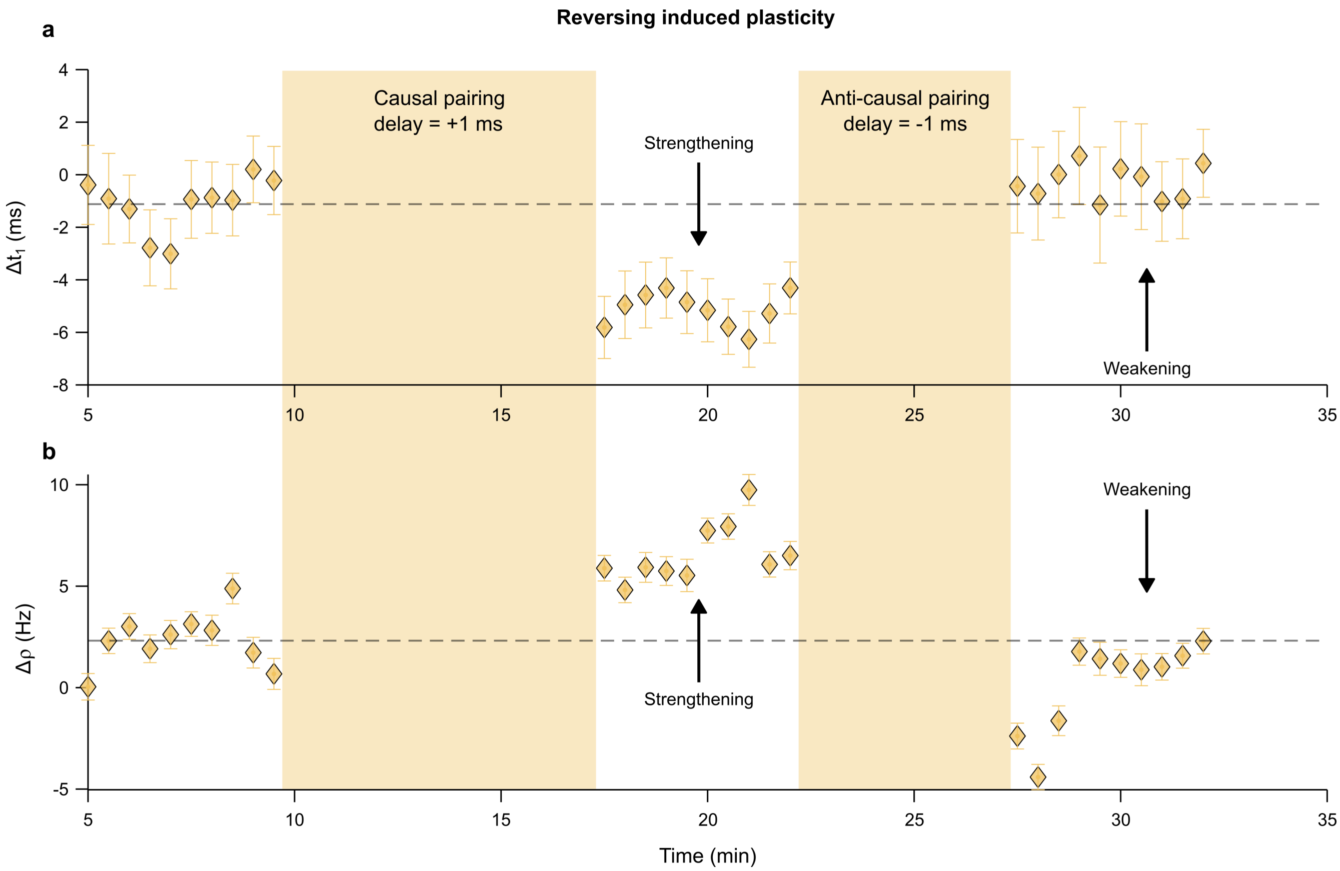

Figure S3: **Reversing induced plastic changes.** Conditional Activity Metrics (CAM) features were computed using 5 min windows with 30 s increments. Each data point was normalized by subtracting the mean CAM value obtained during a baseline period (not shown). The figure shows a representative pair of a primary dissociated rat cortical neuronal culture (DIV 21), illustrating a segment of data from a systematic delay variation experiment (subsection S1.3). Causal pairing with a 1 ms delay led to strengthening, reflected in a reduction in the first-spike latency, Δt_1 (**a**), and an increase in $\Delta\rho$ (**b**). These effects were reversed following the subsequent delivery of an anti-causal protocol (delay of -1 ms). The dashed line indicates the mean ΔCAM levels prior to causal pairing.

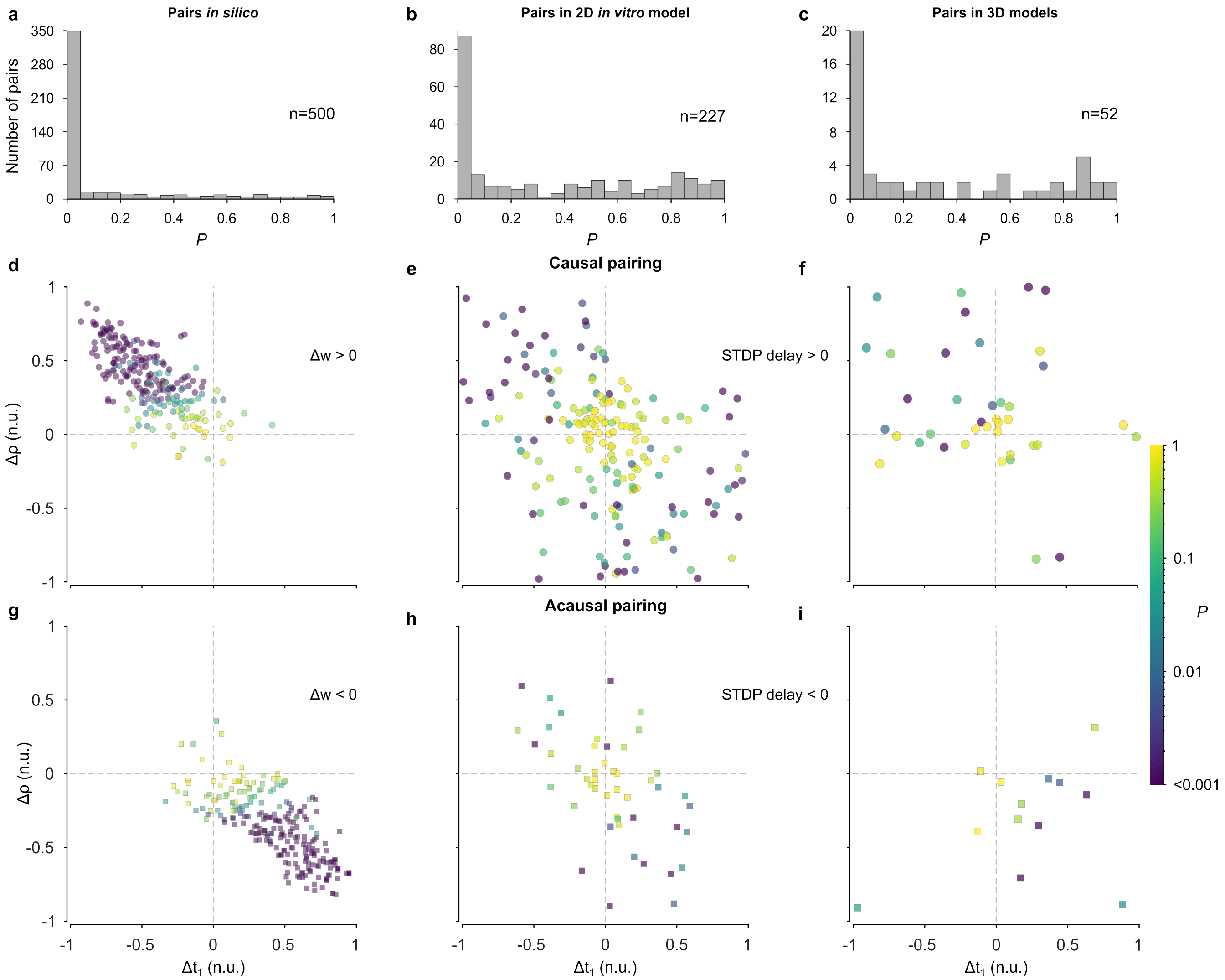

Figure S4: **Summary of pooled stimulated pairs across model systems. (a–c)** Distribution of P-values for stimulated pairs in three models: *in silico* neuron pairs **(a)**, electrode pairs in 2D cultures *in vitro* **(b)**, and 3D *in vitro* and *ex vivo* models (stem-cell-derived organoids and acute mouse slices; **(c)**. Significant effects ($P < 0.05$) were observed in 69.8% of pairs *in silico*, 38.3% in 2D cultures, and 38.5% in 3D tissues. **(d–i)** Normalized ΔCAM values for causal **(d–f)** and anti-causal **(g–i)** pairings. *In silico*, causal pairing shifted ΔCAM into the second quadrant and anti-causal into the fourth, with stronger weight changes producing more significant effects. In 2D networks *in vitro*, results were heterogeneous: following causal pairing we observed shifts into both quadrants, while anti-causal ΔCAM predominantly shifted to the fourth. In 3D models, causal and anti-causal pairings generally showed shifts into the second and fourth quadrants, respectively. Colors denote P-values obtained from a χ^2_2 distribution of the squared robust Mahalanobis Distance relative to the respective control pairs; see section 4 for normalization procedure.

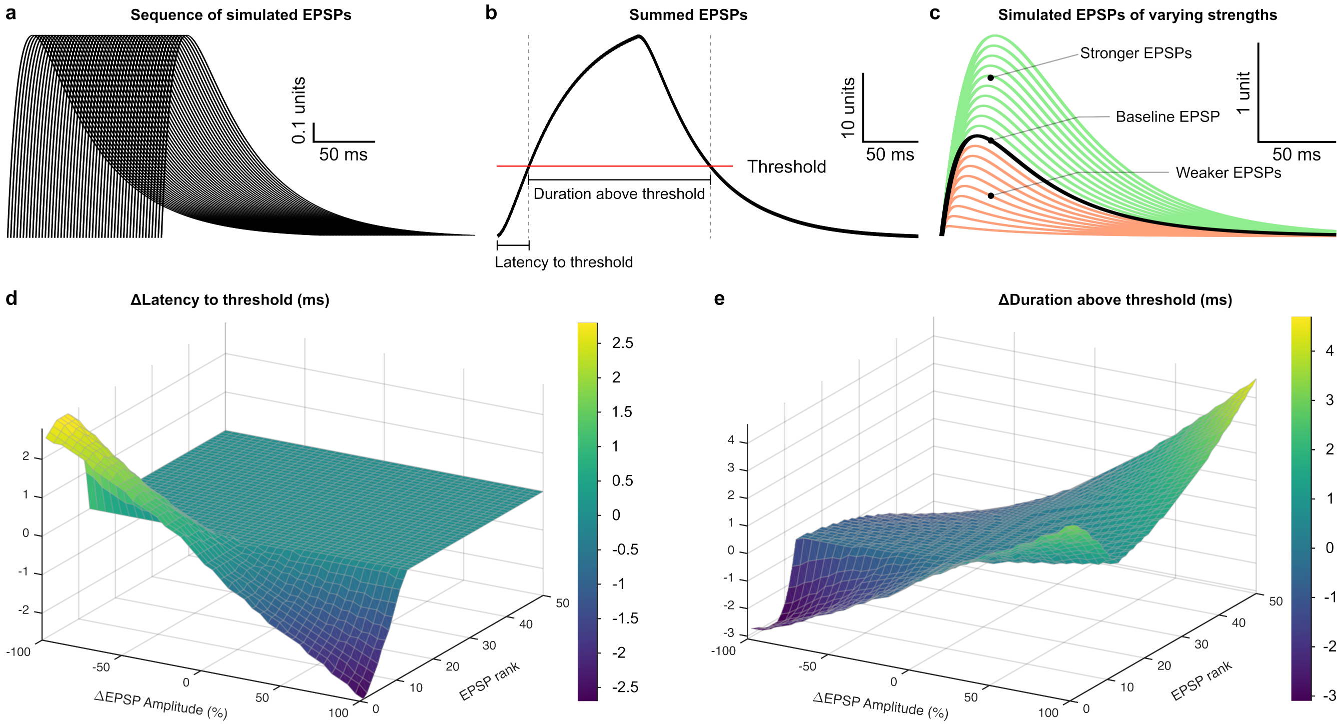

Figure S5: **Nonlinear sensitivity of ΔCAM to integration order and EPSP amplitude changes.** **(a)** A simulated sequence of 50 unit-amplitude EPSPs with a fixed 10 ms inter-spike interval. **(b)** The EPSP sequence was temporally summed at a postsynaptic neuron. A spiking threshold was arbitrarily set at 20% of the summed peak amplitude. **(c)** The baseline summed trace (black) was compared to traces where one of the 50 EPSPs was either potentiated (green) or depressed (salmon). The rising time constant of EPSPs was modeled to be proportional to the peak amplitude. We used the change in latency to threshold (Δt_1) and the change in the duration above threshold ($\Delta \rho$) as estimates for the two CAM dimensions. **(d)** The surface plot shows Δt_1 as a function of the change in EPSP amplitude and its rank in the sequence. For subthreshold ranks (earlier EPSPs), Δt_1 was negatively correlated with amplitude change. This sensitivity rapidly weakened for later ranks, and no changes were observable for suprathreshold ranks. The colorbar and z-axes in **(d-e)** denote the magnitude of change observed in the respective features. **(e)** The surface plot shows $\Delta \rho$ as a function of EPSP amplitude changes and its rank. For subthreshold ranks, the change was positively correlated with amplitude changes. The lowest sensitivity was observed for perithreshold ranks. For later, suprathreshold ranks, the positive correlation was restored. This non-linear relationship reveals that a selective change in synaptic weight (EPSP amplitude) could manifest either as a dual and opposing change in the two CAM dimensions, or a selective change in the second dimension, with ΔCAM being the least sensitive when a perithreshold EPSP was modified.

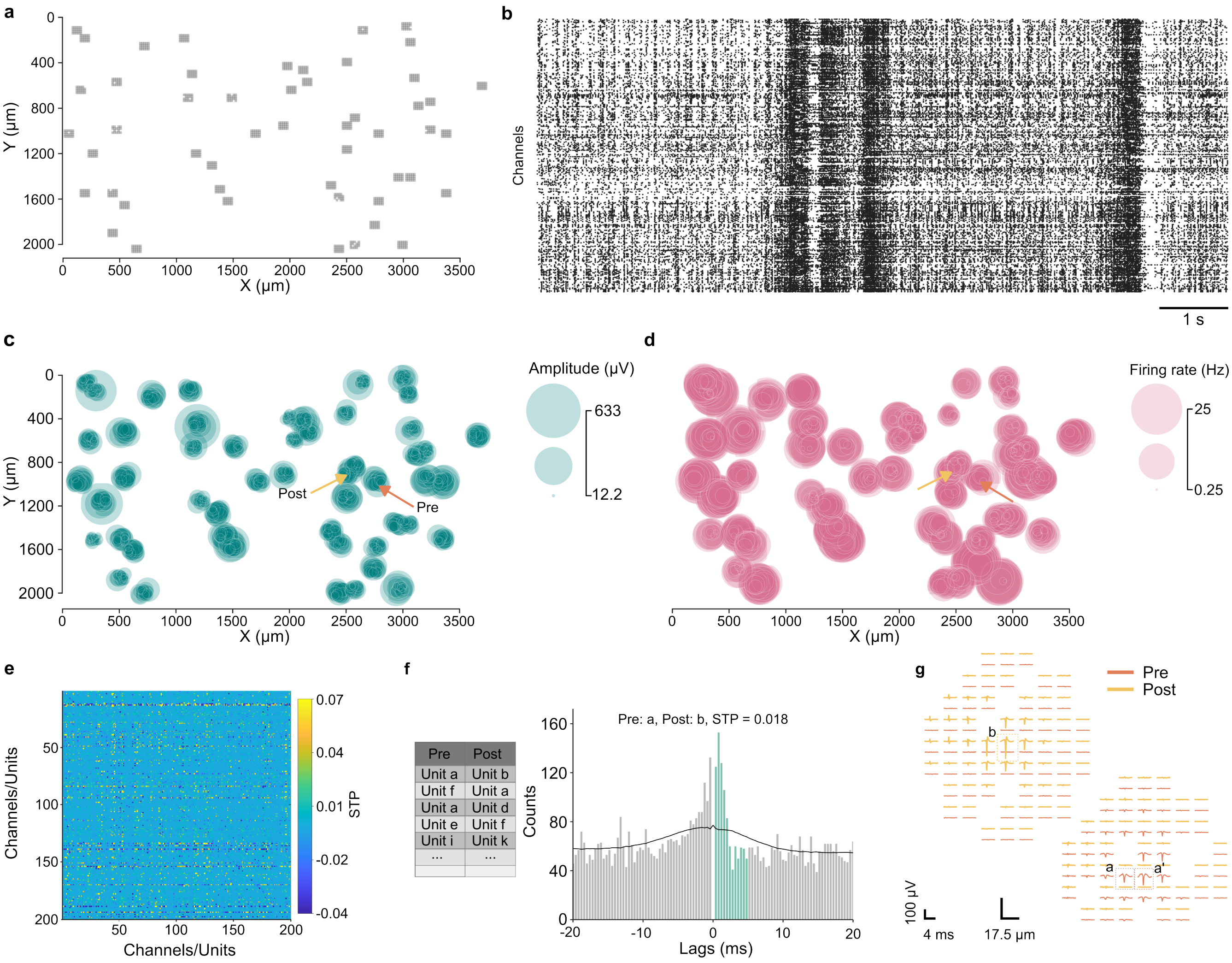

Figure S6: **Pair Selection Procedure (a)** A representative electrode configuration, including up to 1024 electrodes, determined through scans of spontaneous activity. **(b)** Such a configuration was used to record 15 min of spontaneous activity. A representative raster plot illustrates spike times across channels or sorted units. **(c-d)** Spontaneous activity recordings enable an initial pre-selection of candidate electrode/unit pairs. Units with low firing rates and amplitudes (below the first quartile) were excluded. The 200 units exhibiting the highest average amplitudes were retained as putative candidates for subsequent analyses. At this stage, the identities of pre- and postsynaptic electrodes were not yet determined; Pre/Post arrows were added retrospectively for illustrative purposes. **(e)** A matrix of pairwise Spike Transmission Probability (STP) was computed over a window of 0.4-5 ms to estimate putative functional connectivity among the 200 selected candidates. To enhance visualization, the matrix was normalized, after values at the 1st and 99th percentiles were clipped. Based on STP values and cross-correlogram features, candidate pairs were either rejected or categorized into three groups: 'low', 'medium', and 'high' STP. **(f)** Visual inspection was used to further refine candidate selection. Ideal pairs were defined as those exhibiting cross-correlogram with a prominent, short-latency, asymmetric peak, spatial separation of a few hundred micrometers, and no clusters of activity in between pre- and postsynpatic sites. **(g)** The final selection of pre- and postsynaptic sites was informed by spatial footprints. Electrodes with the highest signal amplitude, located in the vicinity of previously selected pre- and postsynaptic electrodes candidates, were prioritized. This refinement aimed to position stimulation sites near the Axon Initial Segment (AIS) to maximize the likelihood of evoking action potentials. For example, electrode a' was selected over a due to its greater amplitude. For clarity, the post-synaptic footprint traces have been vertically offset in the visualization.

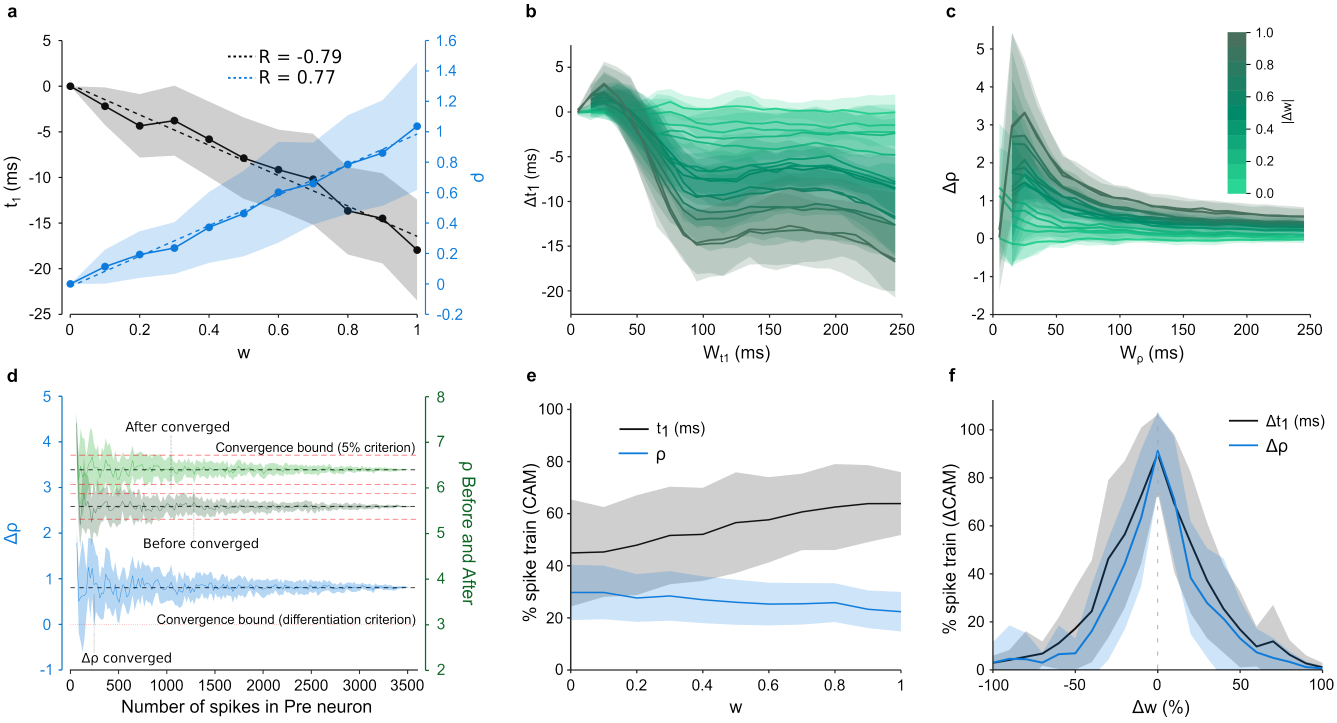

Figure S7: **Further characterization of Conditional Activity Metrics (CAM) (a)** Across simulations, CAM components exhibited robust associations with synaptic strength in network-embedded neuron pairs subjected to systematic variation of synaptic weights. Both parameters, t_1 and ρ, expressed relative to their baseline values at null synaptic weight ($t_1 - t_1(w = 0)$ and $\rho - \rho(w = 0)$), demonstrated statistically significant correlations with synaptic strength (ρ: $r(196) = 0.77$, $P < 0.001$; t_1: $r(196) = -0.79$, $P < 0.001$). **(b-c)** Across simulations, Δt_1 and $\Delta\rho$ were computed as a function of the respective analysis window durations (W_ρ and W_{t_1}), for various $|\Delta w|$. The curves were flipped when $\Delta w < 0$. For all tested Δw, maximal separation was observed for $\Delta\rho$ and Δt_1 with windows $W_{t_1} = [1, 90]$ ms and $W_\rho = [1, 30]$ ms, respectively. Both metrics stabilized at lower absolute values with increasing window lengths, highlighting a trade-off between sensitivity to synaptic changes and the arbitrariness of window selection. **(d)** Example of convergence behavior of ρ and $\Delta\rho$ as a function of the number of spikes in the selected presynaptic neuron, for a synaptic weight change of $\Delta w = 0.5$. CAM values were estimated iteratively by randomly subsampling varying numbers of spikes from the presynaptic neuron's spike train, repetition used to compute mean and standard deviation estimates. CAM was considered to have converged when its values remained within a ± 5% band of the stable value, defined as the mean of the final 20% of CAM estimates. Similarly, ΔCAM was considered to have converged when its values consistently exceeded zero (or were consistently below zero, depending on the metric – t_1 or ρ – and on the sign of Δw). **(e)** The proportion of the presynaptic neuron's spike train required for CAM convergence across different synaptic weights ($n = 1998$, med = 4248, SD = 1270). **(f)** The proportion of the presynaptic neuron's spike train required for ΔCAM convergence across different synaptic weights ($n = 999$, med = 4233, SD = 1269).

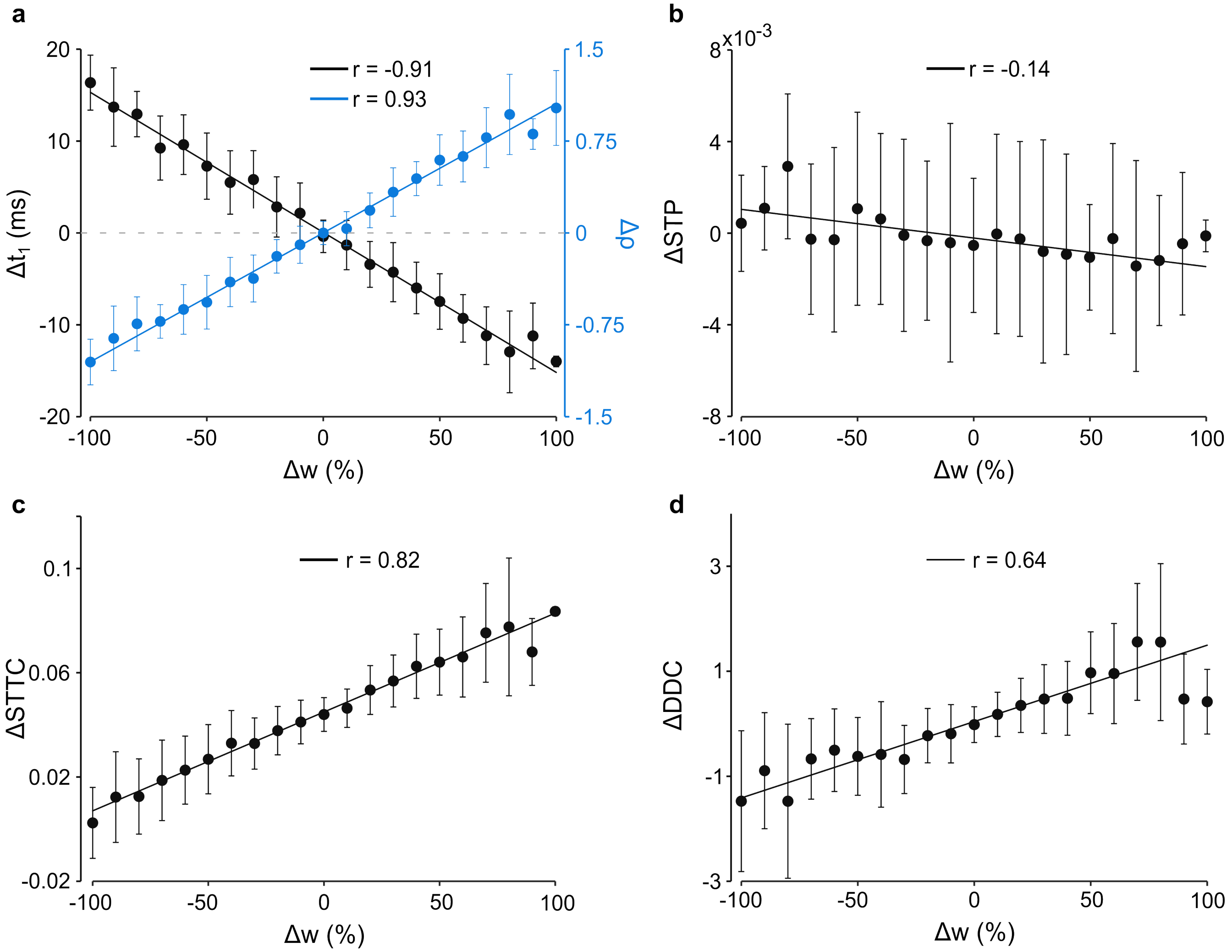

Figure S8: **Comparison of the sensitivity of different connectivity metrics to synaptic weight changes.** **(a)**) Across simulations, both $\Delta\rho$ and Δt_1 exhibited the strongest correlation with weight changes ($r_{\Delta\rho}(498) = 0.93$; $r_{\Delta t_1}(498) = -0.91$, $P < 0.001$), confirming the sensitivity of the metrics to synaptic weight changes. **(b)** The sensitivity of Spike Transmission Probability (STP; [2]) to synaptic weight changes was assessed under identical conditions. Since STP relies on a short temporal detection window for strong monosynaptic connections, post-synaptic spike trains were corrected by subtracting the synaptic delay minus 0.5 ms. Despite this adjustment, STP featured a poor correlation with synaptic weight changes ($r_{\Delta STP}(498) = -0.14$; $P = 0.003$). Similarly, when the short windows were shifted by the synaptic delay rather than subtracted, the correlation remained low. A notable improvement in correlation was only observed when the temporal windows were broadened to tens of milliseconds. **(c)** Spike Time Tiling Coefficient (STTC; [3]) was evaluated under the same conditions and demonstrated a strong positive correlation with synaptic weight changes ($r_{\Delta STTC}(498) = 0.82$; $P < 0.001$). **(d)** Dynamical differential covariance (DDC; [4, 5]) was compared with same conditions and demonstrated a notable correlation ($r_{\Delta DDC}(498) = 0.64$; $P < 0.001$).

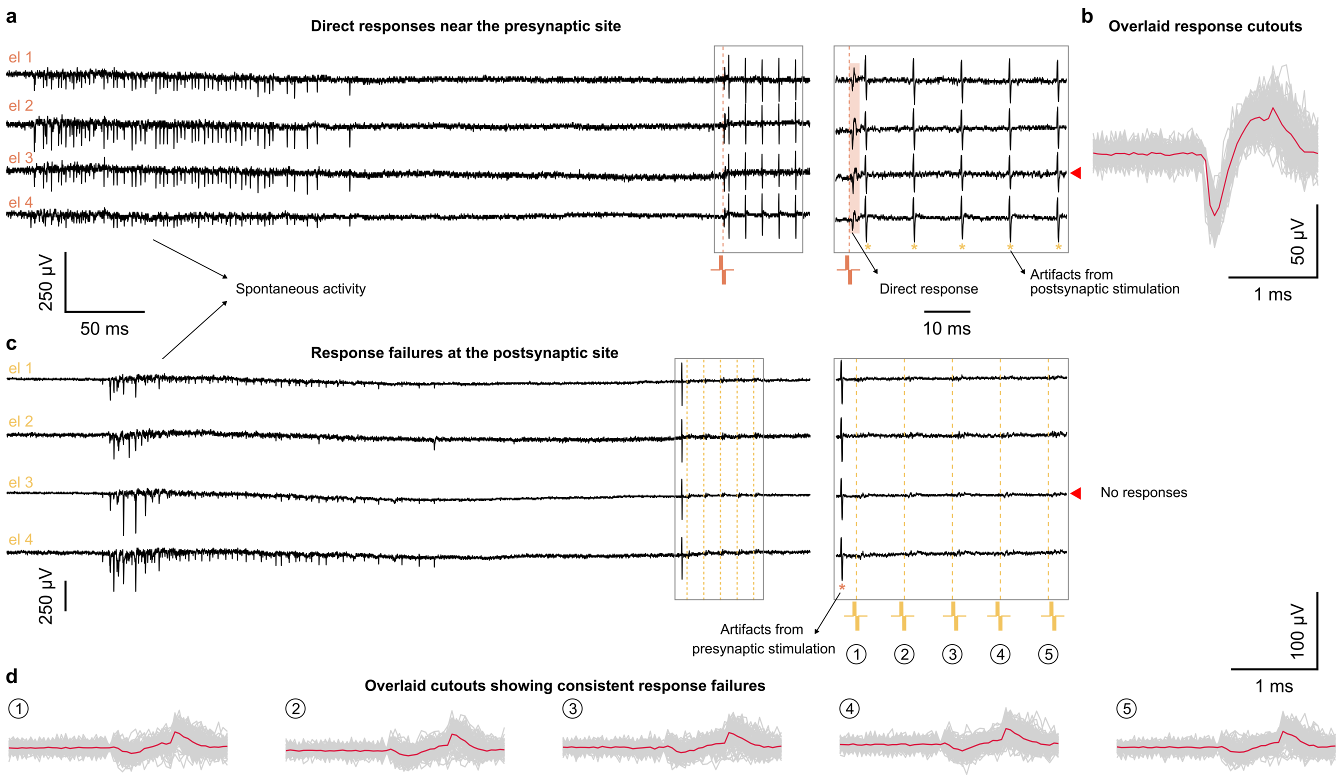

Figure S9: **Illustration of response failures in a pair with insignificant changes in Conditional Activity Metrics (CAM). (a)** Raw voltage traces of four electrodes around the presynaptic stimulation site, cleaned by using our dedicated stimulation-artifact-suppression routine. Spontaneous spiking activity and a single instance of electrical stimulation (red dashed line) are shown. The inset (right) provides a close-up view around the paired stimulation event, revealing a direct neuronal response to the presynaptic stimulus (red highlight) and artifacts generated by the burst stimulation at a distant postsynaptic site (yellow asterisks; 5 pulses at 100 Hz). In this case, artifact suppression was selectively applied only around the presynaptic stimulation times. **(b)** Overlaid response cutouts from 120 repetitions of the paired stimulation demonstrate that direct neuronal responses were consistently evoked at the presynaptic stimulation site. Cutouts were extracted from the trace marked with a red triangle in panel **a**, with the mean response shown in red. **(c)** Raw traces of four electrodes near the postsynaptic stimulation site with artifact-suppression selectively applied around the postsynaptic stimulation times (yellow dashed lines; 5 pulses at 100 Hz, delayed by +2 ms relative to presynaptic stimuli). In the close-up inset, artifacts at the presynaptic site are visible (red asterisk), and all postsynaptic stimuli resulted in response failures. **(d)** Response cutouts of the trace marked with the red triangle in panel **c** (mean traces in red) are overlaid across 120 repetitions of postsynaptic stimulation, showing a consistent absence of direct neuronal responses. Each numbered subpanel corresponds to one of the five stimuli of the postsynaptic burst. No direct responses were observed across all repetitions.

References

[1] R. C. Malenka, M. F. Bear, *Neuron* **2004**, *44*, 1 5.

[2] D. F. English, S. McKenzie, T. Evans, K. Kim, E. Yoon, G. Buzsáki, *Neuron* **2017**, *96*, 2 505.

[3] C. S. Cutts, S. J. Eglen, *J. Neurosci.* **2014**, *34*, 43 14288.

[4] Y. Chen, B. Q. Rosen, T. J. Sejnowski, *Proc. Natl. Acad. Sci. U.S.A.* **2022**, *119*, 24 e2117234119.

[5] T. Kim, D. Chen, P. Hornauer, S. S. Kumar, M. Schröter, K. Borgwardt, A. Hierlemann, Scalable covariance-based connectivity inference for synchronous neuronal networks, Preprint at https://www.biorxiv.org/content/early/2023/06/17/2023.06.17.545399, **2023**.
